# Unrealistic comparative optimism: An unsuccessful search for evidence of a genuinely motivational bias

**DOI:** 10.1371/journal.pone.0173136

**Published:** 2017-03-09

**Authors:** Adam J. L. Harris, Laura de Molière, Melinda Soh, Ulrike Hahn

**Affiliations:** 1 Department of Experimental Psychology, University College London, London, United Kingdom; 2 Department of Psychological Sciences, Birkbeck College, University of London, London, United Kingdom; Technion Israel Institute of Technology, ISRAEL

## Abstract

One of the most accepted findings across psychology is that people are unrealistically optimistic in their judgments of comparative risk concerning future life events—they judge negative events as less likely to happen to themselves than to the average person. Harris and Hahn (2011), however, demonstrated how unbiased (non-optimistic) responses can result in data patterns commonly interpreted as indicative of optimism due to statistical artifacts. In the current paper, we report the results of 5 studies that control for these statistical confounds and observe no evidence for residual unrealistic optimism, even observing a ‘severity effect’ whereby severe outcomes were overestimated relative to neutral ones (Studies 3 & 4). We conclude that there is no evidence supporting an optimism interpretation of previous results using the prevalent comparison method.

## Introduction

People tend to think they are invulnerable and that bad things will happen to others, not to them. These statements represent the dominant position in the literature. Since Weinstein’s [1] seminal paper, a huge number of academic papers (e.g., [2–16]) and popular psychological books (e.g., [17,18]) have expressed this view, ensuring its prevalence not only in social psychology, but also amongst applied practitioners and laypeople (e.g., [19]). Applied practitioners within health psychology, for example, have been concerned that if individuals perceive risks as more relevant to the average person than to themselves, individuals will not take appropriate protective behavior against major risks. It is against this background that Nobel Laureate Daniel Kahneman [17] described optimism as “the most significant of the cognitive biases” (p. 255).

The unequivocality of evidence in favor of unrealistic optimism has, however, recently been questioned. Vosgerau [20] demonstrated that people overestimate the likelihood of positive *and negative* outcomes, relative to the likelihood of neutral ones (see [21] for a failure to replicate Vosgerau’s results with positive outcomes). Other researchers (e.g., [22–24]; see also, [25]) have also demonstrated that the likelihood of negative events is overestimated relative to neutral events. Such findings are difficult to reconcile with the common position that healthy human thought is characterised by a general optimism bias [18,26].

The paradigm that has provided the majority of evidence in favor of a general optimism bias is Weinstein’s comparative methodology [27]. In a typical study, participants are presented with a variety of future life events, and asked to estimate their chance of experiencing each event, relative to the average person. A typical question therefore reads:

Compared with the average student of your age and sex, how likely do you think you are to contract heart disease?

Participants report their answer by circling a number between -3 (much less likely than the average person) and +3 (much more likely than the average person). The logic of the test is that, although each participant’s own risk can be greater or less than the average person’s, the average of all participants’ risks should, by definition, be the average risk. Therefore, if the average response on this scale differs from zero, this is taken as evidence for a systematic underlying bias at the group level. The typical result is that, for negative events, the average score is less than zero. This is taken as evidence of optimism, since we desire *not* to experience negative events.

Although the logic underlying the test is sound, in practice its data are compromised by statistical artifacts. Harris and Hahn [28] demonstrated how seemingly optimistic results could be obtained even from agents who had perfect knowledge about their future, through the mechanisms of scale attenuation and minority undersampling. Moreover, for non-omniscient, but non-optimistic rational agents, base rate regression was another statistical mechanism leading to seemingly biased responses. The detail underlying these mechanisms is provided in [28], but here we provide a brief description of these mechanisms. We then go on to conduct three empirical tests to determine what evidence for comparative optimism is observed when controlling for these statistical confounds.

### Scale attenuation

The most popular scale used in the comparative method is -3 to +3 (e.g., [13–15,27,29–31]). As we show next, problems stem from the fact that for very rare events the sizeable majority of people will be less at risk than the average person. Such events are exactly those most frequently studied in unrealistic optimism research (Welkenhuysen, Evers-Kieboom, Decruyenaere, & van den Berghe (p. 482), for example, grouped risk responses greater than 10% into a single category “because of the low number of responses in these categories” [32]). Where the majority are less at risk than the average person, the minority who are more at risk must choose a positive number on the -3 to +3 scale that is far away from the majority group in order to balance out the responses. In many instances, this will not be possible. To illustrate, we follow [28] and use a thought experiment with perfect predictors (hypothetical participants who know their own future), considering the case of lung cancer, a disease with a base rate/average person’s risk of approximately 6% in the UK [33]. By definition, 6% of the population of perfect predictors know that they will contract the disease. These 6% therefore circle +3 on the response scale, indicating ‘much greater chance than the average person’s.’ The remaining 94% know that they will not contract the disease. Their risk is 0%, as compared to the average person’s risk of 6%. They might (conservatively) circle -1 indicating ‘little less chance than the average person’s.’ The average response is therefore: .06×3+.94×−1=−0.76. This negative difference score, indicating an overall group average slightly below ‘the average person’s risk’ of 0, would be interpreted as optimism by an unrealistic optimism researcher. But note that these data were obtained from agents who *knew* their own level of risk, and responded appropriately on the scale provided to them. The constrained nature of the scale simply does not enable those who will contract the disease to express a high enough comparative risk. For the responses to balance out, those who will contract the disease would have to provide a response that is 15.6 times (0.94 ÷ 0.06) further from zero than the value chosen by those who won’t, which is simply not possible on the scale provided.

Evidence for the influence of scale attenuation in practice comes from [9] who observed a larger comparative optimism effect on a -4 to +4 response scale than a -100 to +100 response scale. This significant reduction in the size of the effect demonstrates a non-trivial impact of the size of the response scale, which in turn impacts conclusions about the size and consequently robustness of an optimism effect (c.f. [34]).

### Minority undersampling

The logic of the comparative methodology is that the average of all individual risks in the population should equal the average risk. We have already seen that a continuous scale is necessary for this to hold, but a further consideration to be highlighted is that things will only average out this way for the population as a whole. Things will also average out this way for a *representative* sample from the population (note, though, the relationship with sample size here: Trivially, an event with a base rate of 1 in 100, needs a sample of at least 100 people to be representative). As soon as the sample is unrepresentative, however, there is no requirement for the average risk of those people in the sample to match the average population risk. Unfortunately, in a random sample taken from a population, it is a statistical fact that the minority is more likely to be undersampled than it is to be oversampled, with the likelihood of undersampling increasing in smaller sample sizes (for implications of this fact in other judgment contexts, see [35–37]). Consequently, were the thought experiment above undertaken with a sample of 100 from the population, it is likely that it would contain fewer than 6 perfect predictors who would actually contract lung cancer. The resulting difference score would consequently be even more negative. For example, were there only 5 individuals who would contract lung cancer the difference score would be .05×3+.95×−1=−0.8. Crucially, minority undersampling suggests that artifactual optimism is possible even if a continuous response scale is provided to participants.

### Away from perfect predictors

In real life, people do not typically *know* whether or not they will experience a particular event. Harris and Hahn [28] demonstrated, however, that the consequences of scale attenuation and minority undersampling still hold for people who have some evidence that allows them to differentiate their own risk from the average person’s (e.g., family history of a disease), and use this to update their own risk in the normatively appropriate manner, via Bayes’ Theorem. The effects are stronger the more knowledge people have to differentiate themselves from the average person, but similar effects hold as long as people have *some* knowledge. Participants likewise do not have to be perfect Bayesians for these artifacts (including base rate regression, below) to assume relevance. They simply need to be sensitive to base rates, possess individuating knowledge, and make use of both in their estimates of personal risk.

#### Base rate regression

**Fig 1:**
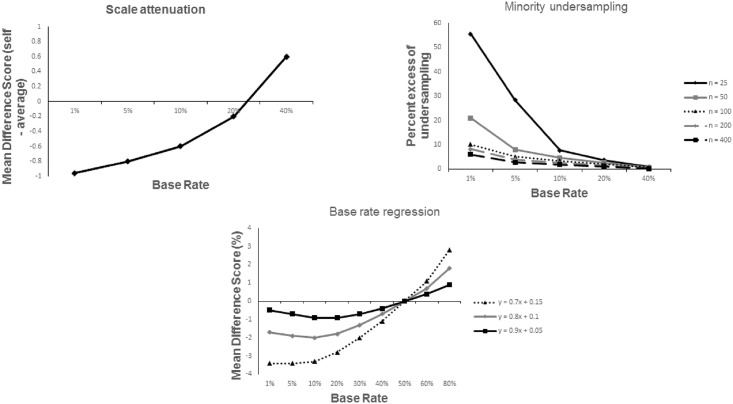
The relationship between event base rate and the three statistical mechanisms (scale attenuation—Top left; minority undersampling—Top right; base rate regression—Bottom). The top left panel represents a situation in which the majority of perfect predictors who won’t get the disease report -1, and the minority who will get the disease report +3. The top right panel shows the excess of instances in which the minority was undersampled relative to the majority—graphing the results for 1 million simulated samples of size 25–400. The bottom panel shows the effect of 3 different levels of base rate regression. Responses are made by predictors who have a result of a test for which a true positive result is 4 times more likely than a false positive result (a likelihood ratio of 4:1), and update their risk according to Bayes’ theorem.

A third statistical mechanism present in such situations is base rate regression. When estimating probabilities, people’s estimates are imperfect. As soon as an estimate is imperfect, unbiased error leads mean estimates to become regressive towards the midpoint of the scale (this stems from the bounded nature of the probability scale). Subsequently, for rare events (those with a base rate < 50%) participants will be prone to overestimate the base rate of the event, whilst they will underestimate the base rate for common events (base rate > 50%). Normative Bayesian updating tells us that estimates of personal risk should be monotonically related to the subjective base rate because best estimates of personal risk combine the base rate with individual diagnostic information via Bayes’ Theorem (e.g., [38,39]). That is, all else being equal, if the subjective base rate increases, so too does the subjective estimate of personal risk. Thus, if the base rate of an event is overestimated, so too will be the best estimate of an individual’s personal chance of experiencing it. For rare negative events, therefore, overestimates (and thus absolute pessimism) follow. Unrealistic optimism is not, however, measured by comparing estimates of an individual’s risk with an objective external standard, because that objective standard is typically unknown. Rather, unrealistic optimism is measured comparatively, using estimates of both individual risk and the average person’s risk (base rate). At a comparative level, base rate regression is likely to gives rise to optimism (see Fig 1, bottom panel). This pattern of absolute pessimism and comparative optimism has been demonstrated in [40]. Note that this base rate regression mechanism is, however, distinct from Moore and colleagues’ differential regression hypothesis (e.g., [40]). As detailed in [28], differential regression concerns differences in the regressiveness of the two estimates for self and average person. The base rate regression phenomenon, by contrast, is about the differences in the predicted distribution of diagnostic knowledge in the world based on real vs. estimated (regressive) base rate (see also [41] for further discussion).

### Implications of the three statistical mechanisms

Given that seemingly biased responses can be obtained from unbiased agents, one cannot infer whether real-world participants are biased or unbiased from the results of studies using the comparison method. People may or may not be rational, but as the results observed by the standard comparison method arise readily from rational agents, these results cannot distinguish between rationality and unrealistic optimism. Harris and Hahn [28], however, identified additional features of the three statistical mechanisms which mean that the artifacts these mechanisms produce have a particular ‘signature’. Fig 1 graphs the results of simulations that vary the base rate of the event being judged, and graph the extent of ‘bias’ introduced by each of the three mechanisms. One can see that each has a systematic relationship with event frequency, such that rarer events give rise to more negative difference scores. Therefore, under what we shall term the statistical artifact hypothesis, the rarer a negative event, the greater is the degree of seeming optimism that should be seen, as has indeed been observed in past studies (e.g., [1,14,27,42,43]).

Our argument thus far has focussed on people’s estimates of negative events, as these constitute the bulk of the unrealistic optimism literature. However, the same argument applies to judgments of the chance of experiencing *positive* events, on the reasonable assumption that very positive events, like very negative events, are rare. Again, the low base rate of extremely positive events implies that most people will not experience the event in question. For positive events, however, this failure constitutes a bad thing, not a good thing. Hence, the statistical mechanisms introduced above that push the group response towards the ‘majority’ outcome will result in seeming *pessimism* for positive events. By definition, this is the opposite of what should be found if people were genuinely over-optimistic about their futures. Consequently, whilst the unrealistic optimism and statistical artifact hypotheses make the same predictions for negative events, they make opposite predictions for positive events.

Unfortunately, studies investigating the possibility of unrealistic optimism for people’s estimates of positive events are far fewer than those investigating negative events. The evidence from those that have included positive events is also much more mixed than it is for negative events (e.g., [44]). Whilst some studies report pessimism (e.g., [40]), a number of others have reported optimism, such that people view themselves as more likely than the average person to experience positive events (e.g., [1,3,11]). However, the statistical artifact hypothesis only predicts unrealistic pessimism for *rare* events. For positive events that are relatively common, the reverse logic applies. For common events, the chance of *not* experiencing them constitutes the rare outcome. Thus, studies that have observed pessimism for rare positive events but optimism for common positive events [43,45] provide direct support for the statistical artifact hypothesis.

The positive events in those studies that have largely found optimism are arguably not rare. Weinstein’s seminal paper [1], for example, used positive events such as “Owning your own home” and “Living past eighty” (p. 810), which seem less rare than the negative events in his study, and consequently the statistical artifact hypothesis would not have predicted pessimism for them. This is supported further by Weinstein’s finding that the perceived probability of the event was the single biggest predictor of participants’ comparative judgments for positive events such that greater comparative responses (interpreted as greater ‘optimism’) were displayed the more prevalent the positive event was perceived to be.

Ratings for perceived probability in [1] came from a separate group of participants, who rated the probability, controllability, stereotype salience and their personal experience with each event. A partial correlation was then conducted between event valence and comparative ratings, resulting in a significant positive correlation, suggesting that comparative ratings were more positive for positive events than negative events, even after controlling for these event characteristics. This result would have been stronger had [1] obtained ratings from the same participants (as we do in Study 1). Secondly, it is unclear from the above analysis whether both the comparative ratings for the negative and positive events remained *optimistic* after controlling for these characteristics, as a significant correlation does not require this result to hold.

Perhaps as a result of the practical implications of the unrealistic optimism phenomenon for negative events, particularly in health psychology, very few subsequent studies have further investigated positive events. Of those that have, some (e.g., [11,46]) have used very similar materials to [1] and, consequently, the same argument is levelled against them. Thus Hoorens, Smits and Shepperd (p. 442) concluded that “researchers have particularly sampled common desirable events and rare undesirable events, the very kinds of events that are likely to produce comparative optimism” [47]. Their own study sought to overcome this limitation by having participants self-generate events; however, the most frequently generated event types in their study were again “variations on themes that typically appear in studies involving experimenter-generated lists of events” (pp. 445–446).

In summary, within the unrealistic optimism literature there is far less evidence concerning positive events, and it is unclear that the sometimes observed optimistic responses for positive events resulted from anything other than their statistical properties—namely that they were much more prevalent than the negative events studied. The few studies that have more fully explored both event valence and event frequency [40,43,45] found comparative responses that are negative for rare events and positive for common events, as predicted by the statistical artifact hypothesis. Given, however, the inconsistencies in the literature, and the importance of these results concerning rare positive events for adjudicating between unrealistic optimism and statistical artifact hypotheses, a replication seems desirable. Furthermore, a new study makes it possible to collect, *from the same individuals* (differentiating it from [1,45]), not only comparative ratings, but also ratings of the critical factors of event frequency and event desirability. Regression analyses can then be used to probe quantitatively whether there is evidence for bias above and beyond the statistical artifacts in the data that scale attenuation, minority undersampling and base rate regression will give rise to. The statistical mechanisms will generate patterns of means indicative of optimism for negative events (and pessimism for positive events) even where participants are not optimistic (or pessimistic). However, this does not rule out the possibility that participants are genuinely optimistic and suitable statistical analyses may detect evidence of such optimism above and beyond the artifactual confounds that plague the comparative method. This was the key original contribution of Study 1.

#### Event controllability

None of the statistical mechanisms could exert any influence if participants did not believe that they had any specific knowledge with which they could differentiate their own personal risk from that of the average person. In the absence of any distinguishing information, everyone should estimate that their own risk is the same as the average person’s (i.e., circle ‘zero’ on a -3 to +3 scale), and hence the average should clearly be zero. The statistical artifacts require participants to have some information that enables them to differentiate their own risk from the average person’s. For example, having a family history of the disease is the strongest predictor of an individual’s lifetime risk of contracting many common diseases [48]. Where individuals perceive an event to be controllable, that provides them with further information that enables them to differentiate their chance from the average person’s. For example, the average person’s risk of cancer is an average across *both* smokers and non-smokers. By not smoking, non-smokers possess diagnostic information suggestive of a lower than average risk of contracting lung cancer by mathematical necessity (of course, a particular non-smoker may have other risk factors that render her overall personal risk higher than the average). Thus, the degree to which an event is controllable can result in different individuals being differentially susceptible to the event, and so perceived controllability *should* moderate the degree of comparative optimism observed. Such a result has been observed in past unrealistic optimism studies using the comparative method (e.g., ([1,14,27,49–51]; see also [52], for a meta-analytic review). We therefore also required participants to estimate event controllability in Study 1.

#### The relationship between the statistical artifact hypothesis and egocentrism

We have already highlighted above that other studies have observed the pattern of results predicted by the Statistical Artifact Hypothesis (e.g., [43,45]). These authors offered their results in support of an egocentrism account for unrealistic comparative optimism. On the egocentrism account, the relationship with event frequency is taken as evidence for the thesis that participants’ comparative estimates reflect insufficient consideration of the average person’s chances of experiencing each event. In this way, participants recognise that they, themselves, are unlikely to experience rare events and likely to experience frequent events. Whilst the same is also true for the average person, participants do not assign this recognition sufficient weight in their comparative judgments. Thus, for example, on the egocentrism account, “comparative estimates for a low base-rate [infrequent] event should be low because people consider their own low likelihood of experiencing the event without fully integrating others’ low likelihood of experiencing the event” ([45], p. 1344). The egocentrism hypothesis also predicts the same role of controllability as the statistical artifact hypothesis (see [45]), since participants underweight the fact that others, as well as themselves, will exploit controllability to reduce their chances of experiencing negative events and increase their chances of experiencing positive events (see also, [12]).

The close relationship between the predictions of egocentrism and the statistical artifact hypothesis is not an accident because data from rational belief updaters might, on first inspection, be interpreted as being egocentric. A simple example reflecting only the parameters aforementioned can illustrate this. Consider an individual who self-reports that they are less likely than the average person to contract Disease X because it is controllable, but that they have the same chance as the average person of contracting Disease Y because it is not controllable. This ‘egocentrism’ is rational on the reasonable assumption that not everyone in the population will exploit the controllability of Disease X. Those individuals who do not take steps to avoid Disease X will push the average risk higher than the risk for those who do take steps to avoid Disease X, in the same way that people with fewer than two legs push the average leg count below that of the majority. An extant empirical question is whether the degree of egocentrism in an estimate exceeds a rationally acceptable amount. Harris and Hahn’s analysis [28] demonstrates that *this* is the evidence required to support an egocentrism account. It is possible that this would be observed—Windschitl and colleagues [53] observed that, although some egocentrism could maximise accuracy in predicting the outcome of two person (self vs. other) competitions, participants were typically overly egocentric in their use of evidence—but it has not been demonstrated thus far in the unrealistic optimism literature.

In addition to the data described above, evidence for egocentrism has been taken from studies that show participants’ comparative estimates to be better predicted by their ratings of their own likelihood than by their ratings of the average person’s likelihood across events [43,45,54]. Such a result is, however, completely uninformative with regard to the information participants are using to make their comparative judgments. A comparative judgment simply calculated as the ratio of personal risk estimate to average risk estimate (see [55]) can readily produce this result with no differential weighting (as recognised in [53]). The reader can verify this for themselves by using the data from [55] (reproduced in S1 Table). Computing a partial correlation coefficient between average risk estimates and the ratio, controlling for self risk estimates, yields a value of .65, whilst the same for self risk estimates, controlling for average risk estimates yields a higher absolute value (-.81). We should not consider a comparative score that is a simple ratio of personal and average risk estimates as representing psychological underweighting of the influence of the average(!). Calculating a comparative score in this way is a perfectly legitimate way for participants to estimate comparative risk. Consequently, the fact that this pattern of partial correlations (supposedly diagnostic of egocentrism) can be generated in this way is an existence proof that undermines the idea that such a pattern provides meaningful support for egocentrism.

Finally, Weinstein and Lachendro [12] found decreased comparative optimism when participants were required to take the perspective of “a typical student.” Such a manipulation enhances the specificity of the comparison target, which can be predicted to decrease optimism on the basis that, for rare events (those with a base rate less than 50%), most individuals, including a typical student, will be less at risk than the average person (see also, [28]), so that the difference between participant and target shrinks.

Given that our interest in this paper lies with testing for the presence of genuine unrealistic *optimism* (i.e., as a motivationally driven bias), as measured by comparative judgments, we do not consider egocentrism (or indeed other cognitive, non-motivational accounts, such as focalism (e.g., [43]), see [56] for a review) further in the remainder. Clearly, egocentrism and the Statistical Artifact account [28] are conceptually distinct (and hence empirically distinguishable), but both are at odds with ‘true optimism’. Rather than distinguish these two accounts in this paper, we thus focus on obtaining evidence for genuine comparative optimism. Consequently, predictions of the statistical artifact account below, are also consistent with egocentrism, but we frequently refer only to the statistical account for ease of presentation.

## Study 1

Our point of departure was to probe for evidence of optimism above and beyond statistical artifacts within the standard paradigm of the comparative method. To this end, it was desirable to make our study ‘representative’ of that paradigm. We therefore based it on Weinstein’s seminal study [1], which has been cited more than 1,750 times (Web of Science, August 31^st^, 2016). Our study was designed to be as faithful as possible to Weinstein’s design; modifications were made only to make it easier to tease apart effects of true optimism and confounding statistical artifacts. Of the 40 life events used, 26 were taken directly from those described in [1] (p. 810), and a further 12 were adapted from the original 42 items in order to update them, remove any ambiguity, ensure their relevance for current UK undergraduate students and, most importantly, create rarer positive events (for example, ‘living past 80’ was replaced with ‘living past 90’). We also added two, putatively rare, positive events not included in [1]: ‘Marry a film star’ and an extra level of starting salary (more than £40,000). The key question was whether we would observe optimism or pessimism for these rare positive events.

### Method

#### Participants

102 female undergraduates (100 is a typical sample size of such studies, and 102 enabled equal distribution of the 6 randomised orders of question blocks—see design), aged 18–24 years (median age = 19), from Cardiff University participated in this study in return for course credit or monetary payment. Only females were included in order to reduce unnecessary variability resulting from gender differences in the desirability of, and susceptibility to, different events. Written consent was obtained from all participants, in line with approval by the Cardiff University ethics committee.

#### Materials

Table 1 shows the events used in this study. The critical experimental question was typical of unrealistic optimism studies. Participants were asked: “Compared with the average female student in your year, how likely do you think you are to…”, and instructions stressed that the likelihood rating was for ‘AT SOME STAGE IN YOUR (THEIR) LIFE’ Responses were made on an 11-point -5 to +5 response scale. In choosing a less attenuated version of the -3 to +3 scale typically employed, our replication is a conservative test of the statistical account for previous unrealistic optimism results.

**Table 1:** ‘Unrealistic optimism’ for future life events.

Event	Mean comparative judgment of own chances vs others' chances	No. of optimistic responses divided by no. of pessimistic responses	Mean perceived frequency
Positive events			
Own own home	1.28 ***	11.17 ***	72.35
Like job after university	0.65 ***	2.39 ***	52.78
Starting salary >£20,000	0.4 **	2.93 ***	53.16
Not spend a night in hospital in 5 years	0.25 ns.	1.39 ns.	53.38
Have a mentally gifted child	0.16 ns.	1.67 ns.	19.62
Visit Amazonian rainforest	-0.12 ns.	0.98 ns.	10.31
Home's value doubles in 5 years	-0.19 ns.	0.80 ns.	25.50
Live past 90 years old	*-0*.*45* **	0.73 ns.	22.58
Maintain a constant weight for 10 years	*-0*.*67* **	0.73 ns.	32.86
Graduate with a first	*-0*.*69* **	*0*.*60* *	25.71
Work recognised with an award	*-0*.*74* ***	*0*.*43* ***	11.39
Last whole winter without being ill	*-0*.*74* ***	*0*.*48* ***	28.91
Receive good job offer before graduating	*-0*.*83* ***	*0*.*24* ***	27.05
Starting salary >£30,000	*-0*.*84* ***	*0*.*31* ***	26.20
Achievements acknolwedged in national press	*-0*.*97* ***	*0*.*27* ***	7.53
Earn >£80,000 in 10 years time	*-1*.*08* ***	*0*.*22* ***	16.24
Nationwide recognition within profession	*-1*.*26* ***	*0*.*23* ***	7.11
Starting salary >£40,000	*-1*.*38* ***	*0*.*16* ***	13.28
Marry a millionaire	*-1*.*52* ***	*0*.*19* ***	4.01
Negative events			
Marry a film star	-1.84 ***	9.43 ***	1.10
Contract AIDS	-1.75 ***	8.86 ***	3.33
Divorced within 5 years of marriage	-1.25 ***	3.93 ***	32.56
Lung cancer	-1.21 ***	4.18 ***	12.57
Have a drinking problem	-0.88 ***	2.15 ***	13.35
Be sued	-0.82 ***	3.06 ***	10.51
Be fired from a job	-0.66 ***	2.83 ***	22.28
Heart attack before 40	-0.65 ***	2.27 ***	6.09
Be unable to have children	-0.07 ns.	0.96 ns.	11.20
Heart attack	0.03 ns.	1.03 ns.	17.48
Have car stolen	0.03 ns.	0.79 ns.	20.19
Out of work for 6 months	0.04 ns.	1.00 ns.	37.27
Be the victim of a mugging	0.09 ns.	0.71 ns.	24.25
Buy a car that turns out to be terrible	0.19 ns.	0.63 ns.	39.35
Realise chose the wrong career	0.20 ns.	*0*.*58* *	34.66
Be the victim of burglary	0.22 ns.	*0*.*39* ***	40.74
Be in bed ill for 2 or more days in a year	0.29 ns.	0.77 ns.	67.06
Forced to take an unattractive job	*0*.*32* *	*0*.*43* ***	49.78
Cancer	*0*.*34* *	0.62 ns.	32.31
Break a bone	*0*.*38* *	0.70 ns.	48.39
Injured in a road accident	*0*.*41* ***	*0*.*26* ***	26.92

ns. = nonsignificant. Italicised means represent significant pessimism.

*p<.05.

**p<.01.

***p<.001.

In addition to the main comparative optimism question, we obtained participants’ ratings of six additional characteristics of the events, resulting in a total of 280 questions for each participant. Three of these characteristics were theoretically motivated to predict differences in unrealistic optimism on the basis of either the statistical artifact account or an unrealistic optimism account: event desirability, event controllability and event frequency. The remaining three were included for exploratory reasons: Event importance, event desirability to the average person, and number of steps taken to approach/avoid the event relative to the average person. As the theoretically motivated questions were able to sufficiently answer the research question, the three ‘exploratory’ blocks will not be discussed further, as they explained no significant additional variance in responses.

To elicit the subjective desirability of each event, participants were required to rate the desirability of each event occurring on a scale from -5 to +5. Perceived controllability was elicited on a 0–10 scale, whilst subjective estimates of event frequency were elicited through asking participants to provide a number in response to the question: “Out of 100 female students in your year, how many do you think will…”

#### Design

A within-participants design was employed. Within each question block, there were four potential orderings of the life events. In each ordering, participants rated positive and negative events alternately and similar questions (e.g. different starting salaries) were not located in close proximity to each other. Participants always completed the comparative optimism question (“Compared with the average female student…”) first, as it comprised the main dependent variable of interest in the study. Six orders of the remaining six blocks were devised such that each block occurred in a different position in each of these six orders and the same blocks were not always adjacent to each other.

### Results

The first step of the analysis was to determine whether our negative and positive events were perceived as such by our participants. Responses to the desirability question led to the classification of 21 events as negative (*p*<.05) and 19 as positive (*p*<.05), by single sample *t*-tests against the scale midpoint (zero). The subjective ratings were as we had expected with the exception of the event ‘marry a film star’ which was judged to be a negative event by our participants. In subsequent analyses we therefore classified this event as negative (although all patterns of results reported below, and their significance, are identical if this event is removed from the analysis).

Table 1 shows the results for both positive and negative events arranged in order of decreasing ‘optimism’, as indicated by the mean comparative judgment. A positive value in the mean comparative judgment column indicates that participants tended to rate their own chances of experiencing the event as greater than average, whilst a negative value indicates that participants rated their chances as less than average.

As a first test of the general unrealistic optimism effect, participants’ comparative judgments of their own chances versus others’ chances were averaged across all negative events. The mean response was -0.32, a result which was significantly below the neutral point (zero), *t*(101) = 4.52, *p*<.001. This demonstrates that, at the group level, participants rated themselves less at risk than the average person from the negative events, replicating the traditional ‘unrealistic optimism’ effect. It should, however, now be clear that this result cannot distinguish between an artifactual explanation and a genuine demonstration of optimism. We next employed the same analysis for the positive events. The results for the positive events matched those for negative events: Participants rated the positive events as *less likely* to occur to themselves than the average person (mean = -0.46), *t*(101) = 5.46, *p*<.001, thus displaying significant ‘pessimism’ at the group level, in line with the statistical artifact hypothesis, but contrary to the predictions of genuine optimism. Our study was primarily based on [1] and yet that study observed optimism for positive events while we observe pessimism. The difference in our pattern of findings can, however, be explained by event rarity; the positive events in the present study were deliberately modified to make them rarer. Indeed, when comparing the results reported in [1] with those in our study, only two directly comparable events show opposite results (significant optimism in [1] and significant pessimism in the current study). The first of these, ‘receiving a good job offer before graduation,’ might be explained by the increase in the number of university graduates between 1980 and 2008, which makes this event rarer in 2008 than it was in 1980. The contrasting results for ‘your work recognized with an award’ might speculatively be related to cross-cultural differences in prevalence (between the US and the UK). Otherwise, there is no conflict between the results of our study and of [1].

In conclusion, (rare) positive events overall elicited pessimism, in line with the statistical artifact hypothesis (or egocentrism) and in opposition to the hypothesis of a genuine optimistic bias.

#### Comparing the effects of perceived frequency and event valence

Looking more closely at Table 1, it is clear that, although the overall analyses clearly replicate the result of seeming unrealistic optimism for negative events [1], the individual events present a much more equivocal pattern. The mean responses for 12 of the 21 negative events are in a *pessimistic* rather than optimistic direction (although only 4 are significantly so). Across all 40 events the means were in an optimistic direction for 14 events, whilst they were in a pessimistic direction for 26 events (*p* = .08 by the binomial test). Such variability across individual events is a common finding in optimism research. To what extent is this variability across events explained by the statistical artifact hypothesis?

Four of Weinstein's original items were not included in this study. These were: "Dropping out of college" (to reduce any extra variance introduced as a result of participants being both first and second year students). "Decayed tooth extracted" and "Having gum problems" (as such events may not be future events for some of the sample), and "attempting suicide" (for ethical reasons).

Events are classified here as positive or negative according to participants' subjective ratings.

As a first test, events were divided into four categories (Positive—rare; positive—common; negative—rare; negative—common). Events were coded as positive or negative on the basis of the classification in Table 1, whilst they were classified as common or rare on the basis of median splits performed on participants’ ratings (Home’s value doubles in 5 years” and “Victim of mugging” were not included in this analysis since they were the median events of each valence in terms of frequency). Only three of the events tested were genuinely common in the sense of a prevalence above 50% (see Table 1). ‘Common’ in these splits is hence a relative term. Although the influence of each individual statistical artifact only reverses once an event’s base rate exceeds 50%, this influence is reduced the closer to 50% the base rate is; moreover, the precise influence of the artifacts can depend on the precise way in which participants use the response scale (see e.g., Fig 1).

**Fig 2:**
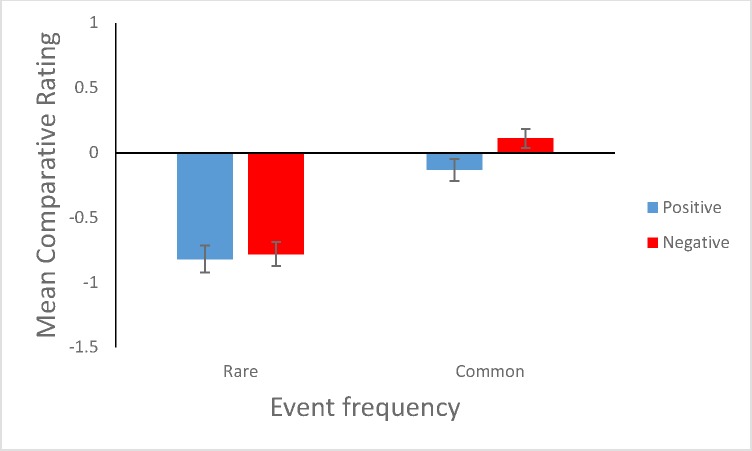
Mean comparative ratings for events according to a 4 way classification on the basis of perceived prevalence and desirability. Error bars are plus and minus 1 standard error.

Fig 2 shows the mean comparative probability judgments for these categories. Common events were viewed as comparatively more likely to occur to the self than the average person than were rare events, *F*(1, 101) = 146.50, *p*<.001, MSE = .43, *eta*_p_^2^ = .59, as predicted by the statistical artifact account (and egocentrism). Notably, no other significant effects were observed in the analysis of variance (ANOVA). In particular, there was no effect of event valence on comparative ratings, *F*(1, 101) = 1.32, *p* = .25, MSE = 1.52, nor was there a significant interaction between frequency and valence, *F*(1, 101) = 3.60, *p* = .06, MSE = .30. The (non-significant) difference in comparative ratings for common positive and negative events (see Fig 2) was in the direction of pessimism (with negative events rated as comparatively more likely for the self than positive events).

#### Regression analyses

That differences in comparative ratings are driven exclusively by event frequency and not by event valence is further suggested by the fact that the two most ‘biased’ seeming sets of comparative responses were for the most neutral items in our data set: Marry a millionaire and marry a film star, both of which had mean desirability ratings that deviated from zero by less than one scale value. This large ‘bias’ is predicted by the statistical artifact hypothesis, because these events were perceived to be the rarest events of their respective valences (see Table 1). It thus seems unlikely that there is any real evidence for unrealistic optimism in this dataset overall. Nevertheless, we also performed a regression analysis as a further check. This analysis also enables us to check whether any evidence for unrealistic optimism might have been obscured by the statistical artifacts. This is the first study to perform such a regression with estimates all taken from the same individuals across both negative and positive events.

If ratings reflect a genuine optimistic bias that represents a kind of ‘wishful thinking’, then one would expect such a bias to increase with the perceived desirability of the event in question. We performed a regression analysis to determine the relative contributions of event frequency, event desirability and event controllability, in predicting the comparative judgments.

After transforming the predictor variables to z scores (see [57] p. 517), we performed a forwards regression. Main effects were added at the first step of the regression, with n-way interactions added at the n^th^ steps. At step 1, two significant predictors emerged in the regression model. As expected, the most powerful predictor was perceived frequency which accounted for 58.4% of the variance in the comparative judgments (beta weight = .56). Event controllability added a further 6% to the predictiveness of the regression model, *F*(1, 37) = 5.89, *p* = .02. At step 2 of the regression, the interaction between event controllability and desirability added 4% (beta weight = 0.16), *F*(1, 36) = 4.74, *p* = .04. This result is also in accordance with the statistical artifact hypothesis: The effect of event controllability should be moderated by desirability (giving rise to the interaction we observed) because increased control has opposite consequences for events of different valence (i.e., approach positive events, avoid negative events). This conclusion was supported by an inspection of the residuals from step 1 of the regression. Moreover, deviations from the best fit regression line were, once again, in the direction of pessimism, not optimism (i.e., positive for negative events and negative for positive events).

No other significant predictors emerged from the regression model. Crucially, desirability failed to capture any variance of its own. Furthermore, the pattern of results was the same if desirability was coded dichotomously (negative or positive) rather than included as a continuous variable, and desirability (either continuous or dichotomous) also failed to predict any variance if controllability was not included in the model. Finally, Table 2 shows that the main conclusions (significant predictive power of frequency and lack of predictive power for desirability) hold in a simultaneous multiple regression, in which the complete model predicts 72% of variance in comparative responses, *F*(7, 32) = 11.60, *p*<.001.

**Table 2:** Table of coefficients from a simultaneous multiple regression predicting comparative responses in Study 1.

Model		Coefficients		t	Sig.
		Beta	Std. Error		
1	(Constant)	-.383	.071	-5.407	.000
	Frequency	.564	.073	7.770	.000
	Desirability	-.064	.079	-.812	.422
	Controllability	-.149	.078	-1.919	.063
2	(Constant)	-.459	.074	-6.197	.000
	Frequency	.511	.072	7.114	.000
	Desirability	-.079	.080	-.993	.328
	Controllability	-.170	.082	-2.083	.045
	Des x Ctrl	.166	.075	2.197	.035
	Freq x Ctrl	-.018	.104	-.174	.863
	Freq x Des	.105	.093	1.131	.266
3	(Constant)	-.443	.077	-5.763	.000
	Frequency	.550	.086	6.422	.000
	Desirability	-.079	.080	-.982	.334
	Controllability	-.158	.083	-1.887	.068
	Des x Ctrl	.156	.076	2.045	.049
	Freq x Ctrl	-.010	.105	-.100	.921
	Freq x Des	.146	.105	1.386	.175
	Freq x Des x Ctrl	-.085	.101	-.843	.406

The above analyses can be considered ‘by-item’ analyses, in that the responses of all participants were averaged for each event, with the regressions being carried out on these average data. Alternatively, one can undertake a by-subjects analysis, with a separate regression undertaken for each participant. Replicating the same findings in a by-subjects analysis suggests that the result generalizes not only across all events, but from the participant sample to the population [58]. Frequency again was a significant predictor of comparative responses (mean coefficient = .28; t[101] = 14.69, *p*<.001). Desirability did not predict a significant amount of the remaining variance in comparative ratings. The mean correlation between desirability and comparative likelihood ratings (after controlling for event frequency—residuals from the regression with frequency were used in this analysis) across individuals was 0.03, *t*(101) = 1.00, *p* = .32). Thus, the comparative ratings appear best explained as stemming from the base rate of the events under consideration, with no influence of event desirability.

**Fig 3:**
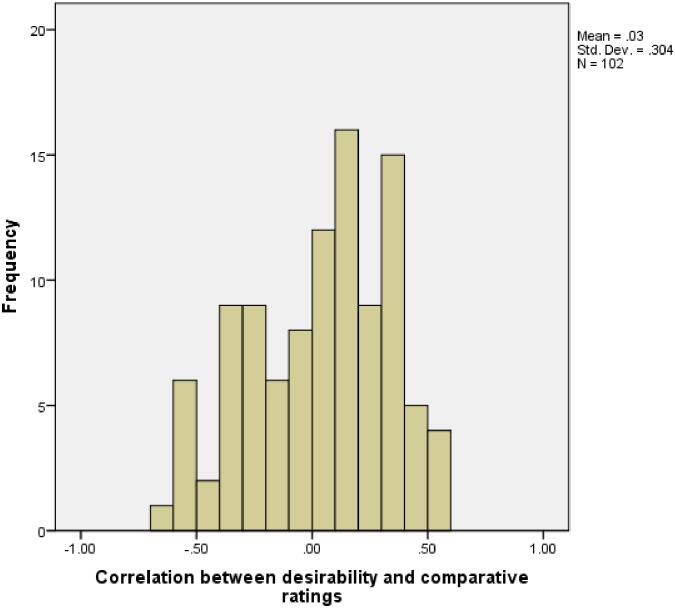
Distribution of the relationship between comparative ratings and desirability ratings (after controlling for perceived frequency) across individuals.

Fig 3 displays the distribution of correlation coefficients from the latter analysis. It demonstrates that some people’s comparative ratings are positively correlated with perceived desirability and others’ are negatively correlated. Remember, however, that the question the comparative method is designed to address is whether there is evidence for optimism at the *group* level. In fact, Weinstein (1980) designed the comparative method for meaningful aggregate level analysis precisely so as to overcome the difficulties associated with studying unrealistic optimism at the individual level (namely, that any individual can have knowledge by which they are either less or more at risk than the average person for a particular event). Whilst a positive relationship between desirability and comparative ratings is necessary for an individual to be comparatively optimistic, it is not sufficient. Some individuals might be more likely to experience positive events and less likely to experience negative events (c.f. [59]). So note that, were all participants to display optimistic responding, it would be indicative of unrealistic optimism at the group level, but nothing can be inferred about the optimism (or otherwise) of individual participants using the comparative method. Crucially, at the group level, as outlined above, there is no overall aggregate level relationship between desirability and comparative ratings.

### Discussion

The aim of Study 1 was to test for a genuine optimistic bias after controlling for the influence of the statistical artifacts identified in [28]. The primary test was whether rare positive events were rated as more likely to occur to the self than to the average person or vice versa. In contrast to the predictions of a genuine optimism account, but as predicted by the statistical artifacts, rare positive events were rated as less likely to occur to the self than to the average person. The weaknesses associated with the comparative methodology predict such data as a result of the statistical mechanisms: scale attenuation, minority undersampling and base rate regression. There could also, however, have been evidence for unrealistic optimism in these results, which was hidden by statistical artifacts giving rise to seeming pessimism for positive events. The way to test for this within the comparative methodology is to perform a multiple regression. In such an analysis, we found that frequency was by far the best predictor of participants’ comparative responses; by contrast, neither desirability, nor event valence were significant predictors.

These findings are in contrast to those of Chambers et al. [45] who reported that event desirability remained significant in such a regression, but they are in line with those of Weinstein [1], who carried out a multiple regression analysis separately for negative and positive events. With all variables entered in the regression, Weinstein did not observe desirability as a significant predictor of comparative ratings for either positive or negative events. Both Chambers et al. and Weinstein, however, regressed comparative ratings from one sample of participants on ratings of event characteristics from a different sample of participants, thus the tests we conduct here are more reliable. Rose et al. [54] obtained both sets of judgments from the same participants, but only for negative (health-related) events. Rose et al.’s results were consistent with those reported here.

The inability of desirability or valence to predict any unique variance in our data speaks rather strongly against recent suggestions that the statistical artifacts identified in [28] exert only minimal influence [34].

Finally, the statistical artifact hypothesis also predicts positive comparative responses for common negative events, and for common positive events. Common positive events were not included, as the predictions of unrealistic optimism and the statistical artifact hypothesis do not disassociate here. Common negative events were not included in our study as they are not typical of unrealistic optimism studies. A small follow-up study using the same method, however, showed positive comparative responses (mean = 0.46, *t*(83) = 3.97, *p*<.001; N = 84 Cardiff University female undergraduates) for seven common, negative events (listed in S2 Table), replicating past findings [40,43,45,54]. This is further evidence in support of the statistical artifact hypothesis and contrary to the predictions of genuine unrealistic optimism.

Harris and Hahn demonstrated via simulation that the flaws associated with the comparative methodology resulted in seemingly biased results being obtained from unbiased agents [28]. Consequently, the comparative method fails a major pre-requisite for an empirical test of bias: results from unbiased agents do not appear unbiased. Study 1 demonstrated that any potential effect of optimism is not strong enough to be observed after controlling for a pattern of results that is predicted by the statistical artifact hypothesis (the variance accounted for by event frequency). Having failed to meet the pre-requisite for an empirical test of bias, it is not appropriate simply to continue to use the comparative optimism method but exert care in relation to the identified statistical artifacts (c.f., [34]). Rather, alternative methods are required to test for comparative optimism; methods that are not susceptible to these artifacts. Studies 2–5 introduce candidate tests.

## Study 2

The inclusion of positive events and the elicitation of judgments of frequency, desirability and controllability, enabling the subsequent multiple regression, represent the best practice one can employ using the standard methodology. In Study 2, we sought to provide a better test of unrealistic comparative optimism. The main difficulties with the standard comparative method stem from the fact that the experimenter has no control over either the frequency of the relevant life events, or the information that participants could and should bring to estimating their own risk. In addition, estimates about real-world events can be influenced by a myriad of factors unrelated to the utility of the events (the availability heuristic of course being the prototypical example of such influence [60]). Such difficulties can be avoided in an experimental test that directly supplies participants with all the information that is (normatively) relevant for their judgments. As long as there is still a need for participants to form an estimate, putative motivational biases underlying genuine optimism have an opportunity to exert an effect.

Such a paradigm can be based on materials and methods used to study the influence of desirability on probability judgments within the judgment and decision making literature. One approach employed in this literature is to provide participants with visual representations of probability information. Participants are then required to estimate probabilities from those representations under contrasting cover stories that manipulate the desirability of the event whose probability of occurrence is depicted. Using such a method, Bar-Hillel and Budescu argued for the ‘elusiveness’ of the wishful thinking effect [61]. Harris and colleagues used such a test to examine the influence of negative utility on individuals’ probability estimates, finding evidence for a ‘severity’ effect, whereby judged probabilities of the same objective probability information were higher when they related to a negative outcome than to a neutral outcome [23]. These types of materials thus already have a successful history of examining biasing effects of outcome utility on probability judgments. The method and materials used in [23] can readily be adapted for a new test of comparative unrealistic optimism.

Studies 2–5 also depart from the methodology of Study 1 in that they do not require participants to directly compare their chances with those of others. A direct comparative methodology was not appropriate since the studies’ control relies on providing the same probability information about both the self and other people. Providing this to participants concurrently would make the identical nature of the information readily apparent, greatly reducing the likelihood of observing any bias in estimates [62]. Consequently, Studies 2–5 are closer in spirit to the ‘indirect’ methodology in comparative optimism studies, whereby participants provide separate estimates of their own chance and the average person’s chance, with bias inferred if there is a reliable difference between these estimates (see e.g., [29,34]). Whilst traditional studies using real-world events can be critiqued on similar grounds to the direct method [28,55,63], the present studies maintain tight control in providing participants with identical information across the two conditions. If traditional unrealistic optimism data reflect a genuine biasing effect of motivation on likelihood estimates, a difference in estimates should be observed between conditions in studies such as these.

**Fig 4:**
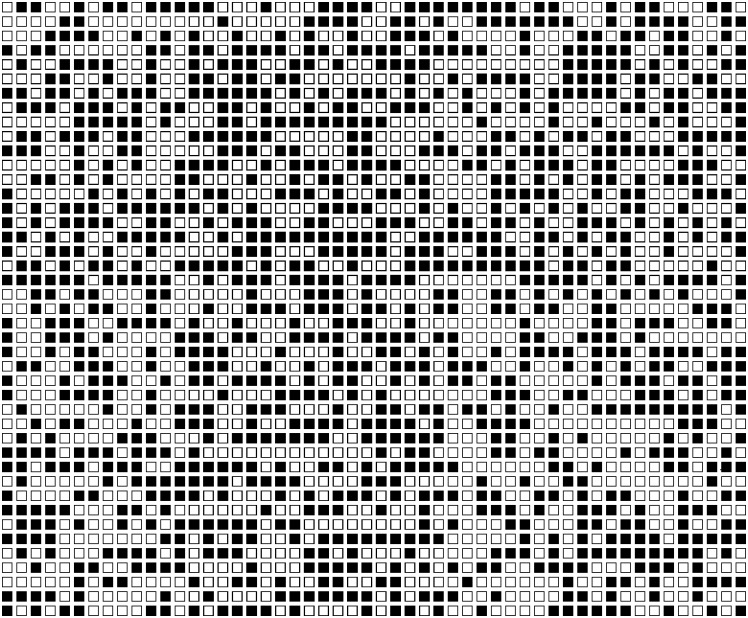
The probability matrix used in the medium probability condition of Study 2.

In Study 2,we used the same visual representations of the probability of a negative event (see Fig 4 below) as in [23], but altered the associated cover story to reflect a potential life event (contracting MRSA on being admitted to hospital), with participants having to estimate that probability either for the ‘self’ or for an ‘average person.’ Given an objective probability that is constant across participants, it would not be possible to explain away any observed effect as a statistical artifact. Study 2 thus constitutes a direct experimental test of the unrealistic optimism phenomenon. Crucial to this design was the fact that participants were supplied with an objective basis for their subjective estimates and that this objective basis was *identical* across the experimental manipulations. To the best of our knowledge, this is the first experiment to test for comparative optimism with a methodology that provides a systematic basis for probability estimates that is consistent across experimental conditions.

### Method

#### Participants

96 Cardiff University female undergraduate students participated in the study in return for either course credit or cash payment. Harris et al. (Experiment 1, Experiment 2) used 100 participants [23]. 96 enabled equal distribution of the six orders of probability levels. Written consent was obtained from all participants, in line with the approval granted by the Cardiff University ethics committee.

#### Design

In order to test the hypothesis that people believe that their chance of contracting a disease is lower than the chances of other people like them (comparative unrealistic optimism), the between participants independent variable was the potential victim (‘target’): namely whether participants were judging their own (‘your’) chance of being exposed to an MRSA-like disease, or ‘Sarah’s’ (a Cardiff University student) chance. The full design was a 2 x 3 mixed design, as participants based their judgments on three different probability matrices, and therefore the within participants variable was the three probability levels: high, medium and low. The dependent variable was the probability estimates, which participants provided by writing a number between 0 (it is impossible that *you* [*Sarah*] will be put in a bed infected by the virus) and 100 (it is a certainty that you [*Sarah*] will be put in a bed infected by the virus). The order in which participants made their judgments using the high, medium, and low probabilities was counterbalanced across participants in each condition.

#### Materials and procedure

In order to completely counterbalance the presentation order of the three probability matrices, six booklet orders were prepared for each condition. A booklet consisted of three pages. Each page repeated the same cover story. The cover stories, which contained the target manipulation, are reproduced below (the words used in the Sarah condition are included in italics):

‘Drug resistant viruses are becoming more and more prevalent in British hospitals. Many of these viruses are potentially deadly and MRSA is a well-known example. At some stage in your*/her* life you*/Cardiff University student Sarah* will be admitted to hospital and unfortunately the prevalence of these drug resistant viruses is showing no signs of decreasing. In the future therefore you*/she* might well find yourself*/herself* facing the following situation. Please read the situation carefully and imagine that it is reality.

You have*/Sarah has* been admitted to a South Wales hospital for a routine procedure. However, an often fatal drug resistant virus is thriving in this hospital. 75% of people who become infected with this virus die from it. This virus contaminates a number of the hospital’s beds. The matrix below represents the distribution of hospital beds infected by the virus (BLACK squares). White squares represent those beds not infected by the virus.

By looking at the matrix below please estimate the chance that you*/Sarah* will be put in a bed infected by the virus (BLACK) thus exposing you*/her* to it.’

The matrix referred to in the text was a black and white probability matrix (see Fig 4). The different probability levels were represented by matrices with different proportions of black cells (5%, 52%, 95%). These matrices were black and white versions of those used in Experiment 1 of [23].

Having completed a consent form and made their way through the experimental booklet, participants were thanked, debriefed as to the purpose of the study and paid (where appropriate).

### Results

One participant was excluded from the analyses as their three probability estimates did not correspond to the basic rank order of the probability levels (the same exclusion criterion used in [23]). After this exclusion there were 95 participants included in the data analysis, 47 in the ‘you’ condition and 48 in the ‘Sarah’ condition.

**Fig 5:**
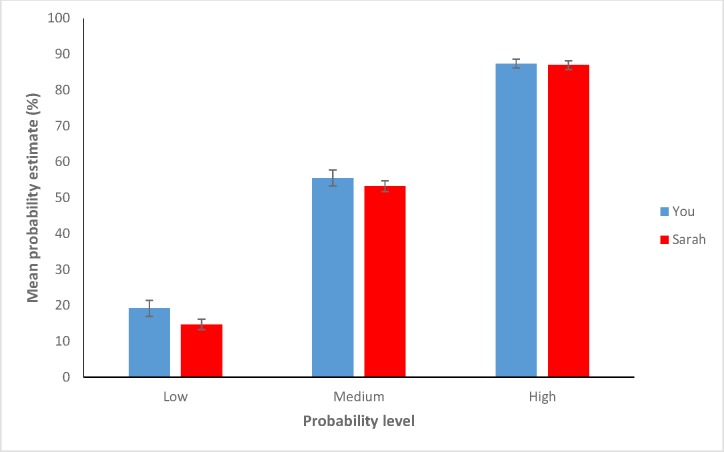
Mean probability estimates made across probability levels by participants in both groups. Error bars are plus and minus 1 standard error.

The probability variable was the only variable to have a significant effect on participants’ probability estimates, *F*(2, 186) = 1151.81, *p* <.001, *MSE* = 101.80. Neither the target manipulation, *F*(1, 93) = 1.958, *p* = .17, *MSE* = 206.02, *eta*_p_^2^ = .02, nor the interaction between the two variables, *F* < 1, attained significance. Examining the pattern of the results (Fig 5), one can see that at each probability level, the (weak) trend was for estimates of self risk to be higher than those of Sarah’s risk—contrary to the predictions of unrealistic optimism. Thus, Study 2 provided no evidence for unrealistic optimism.

The degree of support provided by the data for a hypothesis of unrealistic optimism versus the null hypothesis can be better quantified by means of Bayesian statistical analysis (e.g., [64]). Bayesian analyses allow the direct comparison of the likelihood of observing the data under a specified alternative hypothesis and the null hypothesis. Typically, the null hypothesis is that the effect size is exactly zero, while any value greater or less than this constitutes evidence for the alternative hypothesis. In Study 2, however, the means were in the opposite direction from the predictions of unrealistic optimism. A default Bayesian ANOVA was thus not appropriate in this instance, as it would have examined the evidence that participants in the ‘You’ condition gave higher estimates than in the ‘Sarah’ condition. We therefore conducted Bayesian *t*-tests [64] on each probability level individually. In these tests, we tested a point null hypothesis (effect size is exactly zero) against an alternative hypothesis with a Cauchy distribution that was truncated at zero [65], such that it did not include effect sizes in the opposite direction from optimism. This allows examination of the evidence for the concrete prediction that the probability estimates will be higher in the ‘Sarah’ compared to the ‘You’ condition (unrealistic optimism), versus the null hypothesis that the estimates do not differ between the groups. These Bayesian analyses were conducted using the R package BayesFactor (version 0.9.4), with the package’s default prior values. This is a JZS prior, which for a t-test (used here) has a scaling factor of sqrt2/2 and for an ANOVA (Study 3), a scaling factor of 0.5. Functionally, these priors are equivalent (https://cran.r-project.org/web/packages/BayesFactor/vignettes/priors.html).

Investigating each probability level individually, the data from the low, medium and high probability levels were found to be 11, 8 and 6 times more likely, respectively, under the null hypothesis than under an unrealistic optimism hypothesis (where estimates for Sarah are predicted to be greater than estimates for the self). Following the conventions proposed by Jeffreys (as cited in [64]), these results thus contribute ‘some’ to ‘strong’ evidence for the null hypothesis at the three probability levels. Thus, in Study 2 we observe no evidence for comparative optimism in a design free from statistical artifacts.

## Study 3

Study 2 failed to find any effect in a new comparative optimism test that lacks the problematic features of the ‘standard’ method. Of course, the result simply demonstrates the lack of a difference, and the experiment uses a hypothetical scenario. Against the critique that hypothetical scenarios are simply not sensitive enough to elicit probabilistic biases and thus do not provide very strong tests, it is important to remember that exactly such materials have produced evidence for the influence of outcome desirability on judgments of probability in the past. Moreover, the ‘cover stories’ involved in [23] were arguably less realistic. Specifically, when the ‘bad’ cells in a matrix such as shown in Fig 4 represented ‘fatally poisonous apples’, participants estimated it was more likely that a farmer’s daughter would pick such an apple if she were to pick a fruit at random, than when the ‘bad’ cells represented ‘sour apples’.

In Study 3, we sought to test the generalisability of the null result observed in Study 2, but also to demonstrate a significant result within the same experiment to further demonstrate the strength of the paradigm. Specifically, we tested both an unrealistic optimism prediction as well as an outcome severity prediction (e.g., [20,22–24]). Given our tenet that the strength of the evidence for unrealistic optimism is greatly exaggerated, whilst the severity effect has already been observed in paradigms such as this that are not plagued by statistical artifacts, we expected to find evidence for a severity bias, but not for unrealistic optimism. Such a result would not only provide a replication of the null result observed in Study 2, but would constitute further evidence against a general optimism bias, in that higher probability estimates for more negative events are difficult to reconcile with a position that optimism is a general, persistent human bias. Finally, Study 3 (as well as Studies 4 & 5) recruited both male and female participants.

It should be noted that a severity bias could be tested in two ways. Over- or underestimating the chance of the outcome with respect to the *objective* probability would, in a way, be indicative of a ‘severity effect’ or ‘optimism.’ There are, however, a number of reasons why individuals could over- or underestimate a given probability, many of which will be entirely unrelated to the utility of the event (e.g., the perceptual salience of black vs. white in Study 2). Such reasons will generate over- or underestimates even if the event is not negative. Consequently, a severity effect (that is directly attributable to event utility) is better tested via a *comparison* of estimates across conditions that differ only in their utility—thus controlling for additional factors influencing the accuracy of probability estimates.

**Fig 6:**
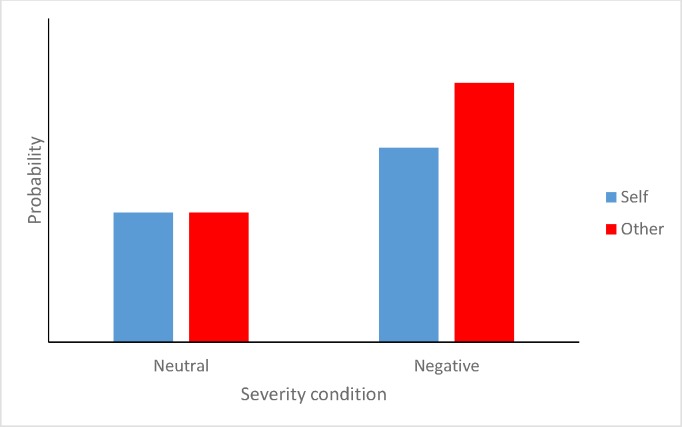
Hypothetical data demonstrating both a severity effect (estimates in the negative conditions are higher than in the neutral conditions) and an unrealistic optimism effect (negative outcomes are less likely for the self than for another).

As in Study 2, we used a paradigm in which an objective probability was defined, available, and constant across experimental conditions. To enhance the generalisability of our results, two new fictional scenarios were created. The scenarios introduced the possibility of a neutral or negative event occurring that participants were told to imagine would either affect them, or would affect another person/other people (‘target’ manipulation). The prediction concerning the severity effect is that participants would provide higher estimates of the likelihood of the severe outcome occurring. The unrealistic optimism prediction is that there will be an interaction between severity and target, such that *lower* estimates will be observed when the negative event will affect participants themselves. Note that we are not setting these predictions *against* each other, as support for both hypotheses could be observed in the current design (see Fig 6).

### Method

#### Participants

Aiming for 400 participants (to provided 50 participants per condition, as in [23]), after deleting subsequent responses from duplicated IP addresses, 389 participants located in the United States (median age = 30; 197 female, 192 male) were recruited via Amazon Mechanical Turk, and compensated with $0.2 for this short experiment. Online consent was obtained from all participants, in line with the ethical approval provided by the (then) Department of Cognitive, Perceptual and Brain Sciences, UCL.

#### Design

Participants were randomly assigned to a 2 (severity: severe vs. neutral) x 2 (target: self vs. other) x 2(scenario: dice vs. container) mixed design (scenario manipulated within participants). The order in which participants saw the two scenarios was randomised across participants.

### Materials

#### Dice scenario

Participants completed an item modified from [20]. In the original studies, participants gambled with real money: they were given $3 and told that they would lose the money, should a specified number of four dice throws show a ‘6’. We modified the task for use on the internet, and also devised a neutral outcome condition. In the ‘self’ condition, the situation was described from a first person perspective, whilst in the ‘other’ condition the main protagonist in the scenario was a third person, “Alex” (shown in italics below). The text provided to participants read as follows:

**Negative Outcome:**
Imagine [you walk / *the poor student Alex walks*] down the street and [you find / *finds*] $100. [You / *Alex*] picked it up, so the $100 is in [your / *Alex’*] pocket. However, a very rich, arrogant and rude person was bending down just as [you / *Alex*] picked up the $100. Seeing a dice for sale in the window of a nearby thrift shop, they propose the following:
[You / *Alex*] will roll a six-sided dice 4 times. If a 6 comes up on at least 2 of these throws, the rich person will get the $100 and [you / *Alex*] will lose the $100. Otherwise [you / *Alex*] can keep it.
What do you think is the chance that a 6 would turn up on at least 2 out of 4 throws, so you / *Alex* would lose the money to the very rich, arrogant and rude person?

**Neutral Outcome:**
Imagine you are / *Alex is* walking down the street with another person. Seeing a dice for sale in the window of a nearby thrift shop, the other person asks you / *Alex* to roll this regular six sided dice four times. What do you think is the chance for a 6 to turn up on at least 2 out of 4 throws?

#### Container scenario

In this scenario, participants in the negative condition were told to imagine the following situation:

A container is to be dropped from the air, and will land somewhere in the area depicted below, with all locations equally likely. The container contains toxic chemicals which are fatally poisonous to humans. Below, you see the area where the container could land. The blue lines are an underground watercourse, which supply drinking water to your city. The red circle indicates the size of the area where toxic chemicals will be released. If this area overlaps at all with one of the water veins, the chemicals will be released into the drinking water, killing thousands of people in your city.
What is the likelihood that the container lands so that it overlaps with one of the water veins, thus poisoning your city’s drinking water and killing thousands?

**Fig 7:**
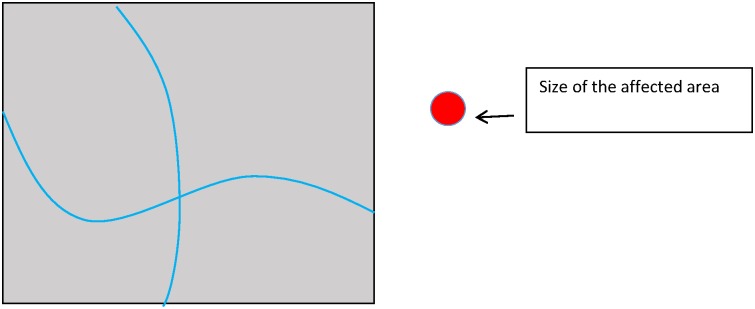
Probability display used in the “container” scenario.

In the neutral condition, participants were told that the container contains organic materials that pose no risk to people or the environment and an overlap between the container and a water vein would cause the drinking water of the large city to taste very slightly different. Moreover, the target manipulation was operationalised through referring to a “European city” rather than to “your city” in the ‘other’ condition (recall that the participants were all located in the U.S.). The ‘area’ referred to in the text is shown in Fig 7.

All responses were provided on a sliding scale from 0 (absolutely impossible) to 100 (absolutely certain).

#### Procedure

As part of a separate project, and unrelated to the current aims, participants first completed the five item private body consciousness scale [66]. Afterwards, in a randomized order, participants completed the dice and container scenarios. Finally, participants completed manipulation checks for severity (e.g., “How bad would it be if at least 2 sixes are rolled”) and target (e.g., “how much would you be affected if at least 2 sixes are rolled?”) on 7-point scales ranging from 1(not at all bad / not at all affected) to 7 (very bad / very affected). Finally, participants were thanked and debriefed.

### Results

#### Manipulation checks

Participants judged that the focal outcome would be worse if it occurred in the severe condition than the neutral condition for both the dice, M_Negative_ = 4.65 (SD = 1.84) vs M_Neutral_ = 1.25 (SD = 0.73), *t* (387) = 23.86, *p*<.001, and container, M_Negative_ = 6.57 (SD = 1.07) vs M_Neutral_ = 3.07 (SD = 1.64), *t*(387) = 25.02, *p*<.001, scenarios, demonstrating the success of this manipulation. Likewise, the target manipulation was successful, with participants indicating that they would be more affected by the outcome in the self than other condition for both the dice, M_Self_ = 3.57 (SD = 2.39) vs. M_Other_ = 1.48 (SD = 1.18), *t*(387) = 10.95, *p*<.001, and container, M_Self_ = 4.64 (SD = 2.18) vs. M_Other_ = 2.65 (SD = 1.93), *t*(387) = 9.55, *p*<.001, scenarios.

#### Main analyses

**Fig 8:**
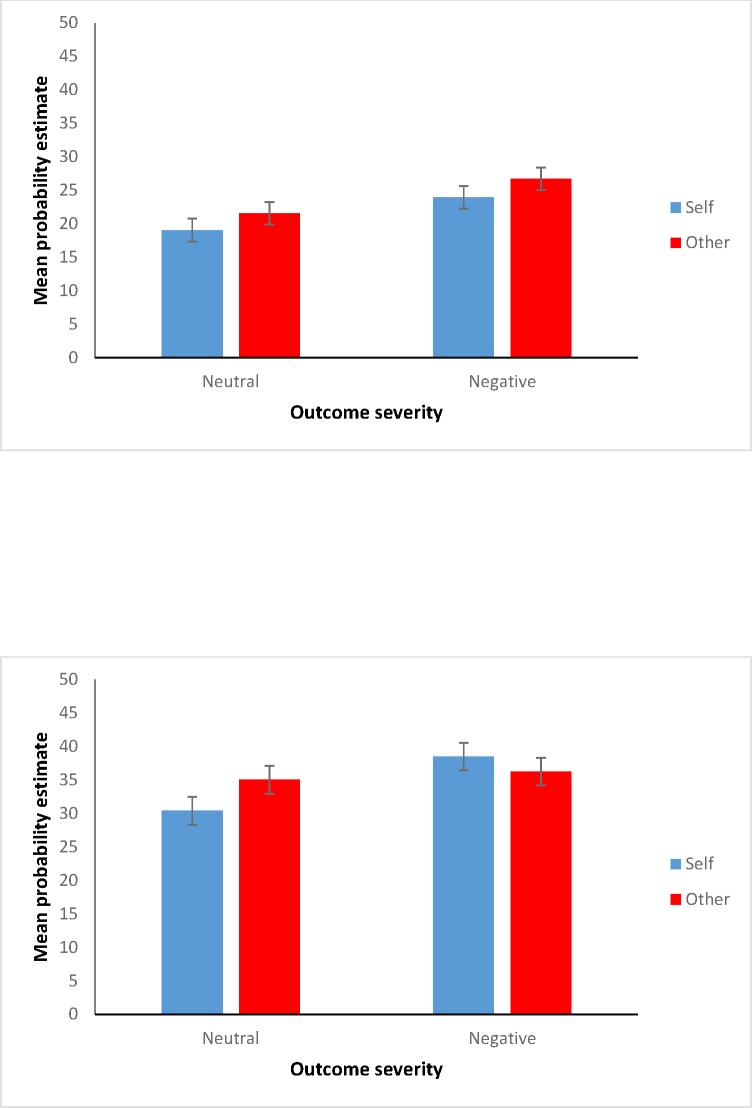
Mean probability estimates in Study 3. The top and bottom panels show data for the dice and container scenarios respectively. Error bars are plus and minus 1 standard error.

Fig 8 shows the data from the dice and container scenarios separately. A repeated measures ANOVA with dice and container scenarios as within-, and severity and target as between-participants factors revealed a significant main effect of scenario, *F*(1, 385) = 124.54, *p*<.001 (dice scenario, mean = 22.85, SD = 16.88; container scenario, mean = 35.08, SD = 20.58). Of more interest, the main effect of severity was also significant, *F*(1,385) = 9.87, *p*<.01. The main effect of target, *F*(1,385) = 1.54, *p* = .22, and the interaction between target and severity *F*(1,385) = 1.13, *p* = .29, were both non-significant. There were no significant interactions with scenario, all *p*s > .10. The interpretation of these inferential statistics is strengthened by means of a Bayesian equivalent of an ANOVA [67], which we were able to use in this instance as the direction of means for the target manipulation was in the direction of optimism (which was not the case in Study 2). The Bayesian analysis (unsurprisingly) showed a clear effect of scenario. We are interested in the predictive power of explanatory models that include the factors of severity and target, over and above the explanatory power of a model solely including scenario. The model including severity was 13 times (‘strong evidence’) more likely than the model only including scenario. However, a model consistent with unrealistic optimism, including self and the self x severity interaction term, was 11 times (‘strong evidence’) *less* likely than the model only including scenario. Finally, and critically, the data were 10 times (‘strong evidence’) more likely to have arisen from the model only including severity and scenario than they were to have arisen from the full model that also included self and the self x severity interaction term.

In sum, we observe no evidence to support unrealistic comparative optimism in people’s probability estimates. In fact, probability estimates of negative events were higher than those for neutral events (see also, [22–24]).

## Studies 4 & 5

Studies 2 and 3 provided a direct test of the unrealistic optimism hypothesis in a controlled experimental design. Despite the presence of a significant severity effect in Study 3, there was no hint of optimism. In other words, the hypothetical materials were sufficiently involving to participants to generate significant effects of outcome utility on judgments of probability; yet, there was no evidence for optimism. The proponent of unrealistic optimism must then argue that unrealistic optimism would only manifest in the presence of genuine, self-relevant outcomes (i.e., in non-fictional scenarios). Study 4 thus aimed to create an experimental situation that was as controlled as those in Studies 2 and 3, but with the addition of a genuine cost to participants. The design was identical to Study 3, and a first-person, non-fictional scenario was created based on the rationale of the dice game used in Study 3. Study 5 employed the same rationale as Study 4, but improved the methodology after the ‘target’ manipulation check failed in Study 4, so as to ensure the believability of the set-up.

### Study 4 method

#### Participants

200 participants (115 female, 85 male; mean age = 23.03, SD = 5.34) were approached on the campus of University College London by an experimenter blind to the hypotheses of the study. Online consent was obtained from all participants, in line with ethical approval as granted by the (then) Department of Cognitive, Perceptual and Brain Sciences, UCL.

#### Design

Participants were randomly assigned to a 2 (severity: negative vs. neutral) x 2 (target: self vs. other) between-participants design.

#### Materials and procedure

Participants were informed that the study concerned participants’ perceptions of games. In the ‘self’ condition, participants were told that they were going to play a game now, whereas in the ‘other’ condition, participants were explicitly told that they would not play the game themselves. First, participants were given verbal instructions about the game.

In the ‘negative self’ condition, participants were handed £5 (holding an endowed object has been shown to increase perceived ownership [68]) and the experimenter (who was blind to the experimental hypotheses) put 6 differently coloured counters (one of which was red) in a bag. It was explained that the participant was going to draw 4 counters from the bag, whilst always placing the counter back inside the bag after each draw. Participants were informed that if they were to pull out the red counter on at least one of the 4 draws, they would be asked to give the £5 back, otherwise they could keep the money. In the ‘negative other’ condition, participants were shown the £5 but were not handed the money. The instructions were identical but rather than directly addressing participants, the game was described from the perspective of “a player of this game”. In the neutral conditions there was no reference to the £5, and therefore no outcome was attached to drawing the red counter.

Following these verbal instructions, participants read descriptions of the game again, presented via the software Qualtrics on an iPad, and were told that we would like them to answer some questions about the game. In all conditions, participants were then asked to estimate the chance that they (or the player of this game) would draw the red counter at least once. Answers were given on sliding scales from 0–100, again on the iPad. Following, as a manipulation check, participants answered the two questions “how bad would it be if at least one red counter was drawn” (not bad at all—very bad) and “how much would you personally be affected if at least one red counter was drawn” (not at all—very much) on 7-point scales.

### Study 5 method

As previously mentioned (and outlined below), the manipulation check questions for Study 4 suggested the target manipulation failed. In addition, the experimenter (MS) observed that many participants acted as though to return the £5 after ‘not losing’, and were surprised that they were able to keep it. This potentially compromised the real nature of the incentives in this experiment. Study 5 was designed as a replication of Study 4, whilst addressing this issue.

#### Participants

240 participants (136 female, 104 male; mean age = 23.78, SD = 6.66) were approached on the campus of University College London. Written consent was obtained from all participants, in line with the ethical approval granted by the (then) Department of Cognitive, Perceptual and Brain Sciences, UCL.

#### Materials, design and procedure

Unless specified, details are the same as in Study 4.

The main change was to the consent form for the study. Participants in the severe-self condition received an additional consent form, which was intended to ensure that they believed the £5 was really theirs. Participants were approached and asked if they could spare a few minutes for a research study. If they agreed, they were handed an envelope and asked to take out the large (A4) piece of paper that was inside it. This piece of paper was the consent form printed with an official UCL letterhead. The procedure also served to ensure that the experimenter was blind to the participant’s experimental condition (the experimenter was no longer blind to the study hypotheses in Study 5).

In addition to the standard information to ensure informed consent, participants read the following passage that was highlighted by having a box drawn around it:

“In social research, it is beneficial for the experimenter not to know everything about your experience, so as to prevent them from being able to inadvertently bias the results of a study. We have therefore hidden some information about this experiment on the inside of the envelope you have been given. Please do not tell the experimenter what you see there. You should find a £5 on the inside of the envelope. The £5 note is now yours. You might, however, lose the £5 depending on the outcome of the game that will be described to you on the iPad. You should also find your participant number is written on the inside of the top of the envelope. You will need to enter that when required on the iPad.

Please do **NOT** pass this piece of paper to the experimenter.”

Outside the box, the text continued:

“Please sign to indicate that you understand this information and consent to participate in the study. By consenting to participate, you understand that the £5 is now yours, UNLESS you lose it in the game that will be played.”

Underneath this text, the left side of the paper requested a signature and a date, whilst the right side thanked the participants for their assistance, and was signed by AJLH who was identified as the lead investigator. The text in all other conditions was the same, except the text “You should also find a £5…depending on the outcome of the game that will be described to you on the iPad” was omitted.

The target manipulation check consisted of questions that allowed us to focus our analyses by excluding participants who had not understood the descriptions of the study. These were tailored to the severity condition participants were in. In the neutral condition, participants were simply asked “Who will play this game?” In the severe condition, participants were asked this, as well as: “Who will lose £5 if the red counter is drawn at least once?” and “Who will be negatively affected if the red counter is drawn at least once?” The choice options for all these questions were “Me” and “Someone else.” In order to avoid asking participants any seemingly non-sensical questions, only participants in the negative condition were asked how bad it would be if at least one red counter were drawn.

### Results (Studies 4 & 5)

#### Manipulation checks

In Study 4, the severity manipulation was successful. Participants in the negative condition indicated that the outcome was worse (M_negative_ = 3.86, SD = 2.11), compared to participants in the neutral condition (M_neutral_ = 1.79, SD = 1.34), *F*(1,196) = 72.15, *p*<.001. However, there was also a significant main effect of target, with participants in the other condition reporting that they would find the outcome (across severity conditions) worse (M_other_ = 3.24, SD = 2.11) than participants in the self condition (M_self_ = 2.41, SD = 1.9), *F* (1,196) = 11.6, *p*<.01. The interaction between target and severity did not reach significance, *F*<1.

The answer to the question of how much participants would be personally affected if at least one red counter was drawn suggested, however, that the target manipulation was not successful in Study 4. No difference was observed between the target conditions, *F*(1,196) = 2.34, *p* = .13 –participants in the “other” condition gave slightly higher ratings as to how much they would be personally affected (M_other_ = 2.45, SD = 1.8) compared to participants in the “self” condition (M_self_ = 2.08, SD = 1.64). However, there was a marginally significant main effect of severity, in that participants in the negative condition gave higher ratings (M_negative_ = 2.49, SD = 1.71) than participants in the neutral condition, (M_neutral_ = 2.04, SD = 1.72), *F*(1,196) = 3.47, *p* = .06. The interaction between target and severity was not significant, *F*(1,196) = 1.86, *p* = .17. Despite the seeming failed manipulation in Study 4, we note that it *was* the case that the participant would be affected by the outcome in the ‘self-negative’ condition and not in the ‘other-negative’ condition. Consequently the failed manipulation check is rather suprising, and it is plausible that this failure may have lain with the manipulation check question rather than the manipulation itself. We therefore continue with our analyses of the probability estimates, but addressed the failed manipulation check in Study 5.

In Study 5, responses from 200 participants were originally collected. Using the “Who will play this game?” question as a filtering device, 32 participants were excluded, predominantly from the ‘other’ condition (27 participants). So as to avoid large inequalities in cell sizes across conditions, 40 further participants were recruited (38 ‘other’; 2 ‘self’–note that the experimenter was still blind to the experimental condition and the distributions of the conditions for these ‘top-ups’; the same significance and descriptive patterns were observed in the results if these participants are excluded from the analysis). The patterns of results are the same whether no exclusions are made, exclusions are made only on the single manipulation check question, or if participants are only included if they answered *all* their manipulation check questions correctly. We here present the analyses with the latter exclusions in place, which led to 189 participants being retained for analysis (Self/Neutral: 47; Self/Negative: 42; Other/Neutral: 52; Other/Negative: 48).

#### Probability estimates

**Fig 9:**
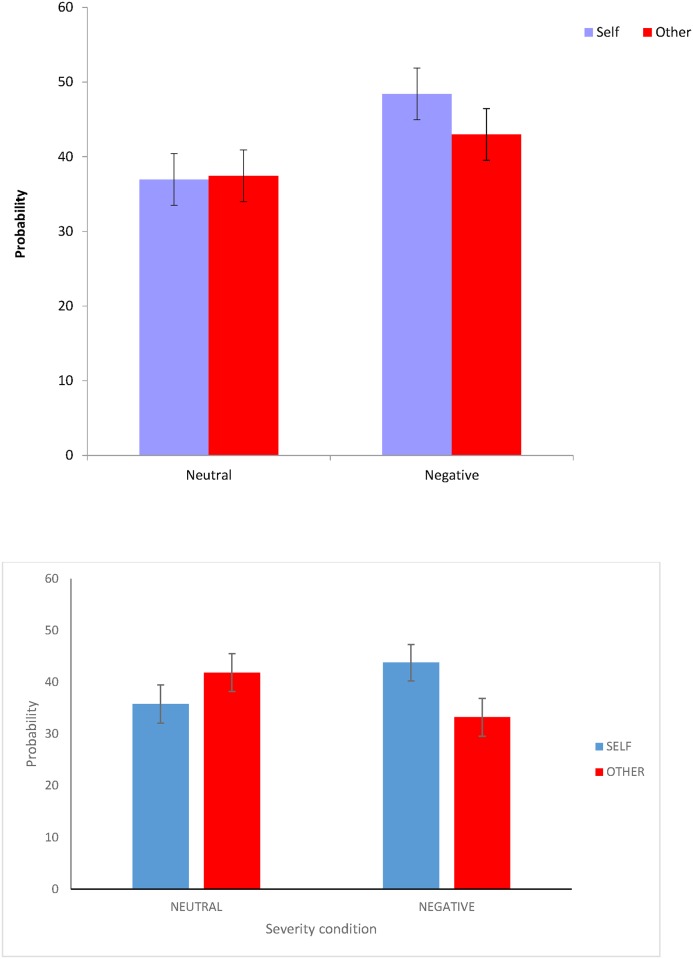
Mean probability estimates across the self and severity conditions in Studies 4 (top panel) and 5 (bottom panel—After excluding participants who failed any of the manipulation checks). Error bars represent one standard error of the mean.

Estimates are shown in Fig 9. Whilst the precise pattern of results differs across Studies 4 and 5, inspection of responses to the negative condition demonstrate that neither study supports an unrealistic optimism hypothesis, which would predict lower estimates for self than for other with negative outcomes (e.g., Fig 6).

In Study 4, a main effect of severity was observed, *F*(1,196) = 6.03, *p* = .015, with participants in the negative condition providing higher probability estimates (M_negative_ = 45.7, SD = 25.74) compared to participants in the neutral condition (M_neutral_ = 37.2, SD = 23.05). There was no effect of the target, *F*<1, *ns*. Moreover, there was no interaction between severity and target, *F*<1.

As suggested in Fig 9, the pattern of results was different in Study 5, where the only significant effect was the severity x self-relevance interaction, *F*(1, 185) = 5.60, *p* = .019, eta_p_^2^ = .03 (all other *F*s < 1). Simple effects demonstrated that there was no effect of the target manipulation when the outcome was neutral, *F*(1, 185) = 1.57, *p* = .21. When the outcome was severe, estimates for the self were *higher (i*.*e*. *pessimistic)* than for another, *F*(1, 185) = 4.30, *p* = .04, thus the interaction term provides no evidence in support of the unrealistic optimism hypothesis.

In order to strengthen the results given by inferential statistics, we again considered running the Bayesian equivalent of an ANOVA. However, in both studies, the probability estimates of participants in the self condition in the negative condition were actually higher than the estimates of participants in the other condition, and are thus in the opposite direction to what an unrealistic optimism account would predict. Therefore, to examine the evidence for the concrete prediction made by an unrealistic optimism account that the probability estimates will be higher in the “other” than in the “self” condition in the negative condition, we tested the null hypothesis for these conditions against an alternative hypothesis that was truncated at zero in a Bayesian *t*-test [65], as in Study 2. The data were found to be 9 times (approaching “strong” evidence—Study 4) and 11 times (“strong evidence”) more likely under the null hypothesis than under the unrealistic optimism hypothesis

### Discussion

The overall patterns of results reported were different in Study 5 vs. 4. A feature both experiments did, however, have in common was that neither of them showed any evidence of optimism. Comparative optimism should manifest itself in lower estimates for the self than another individual in the negative condition. Such results were not observed in either of these studies or in Studies 2 or 3.

We have no explanation for the difference in the pattern of results between Studies 4 and 5. An inspection of Fig 9 suggests that the significant interaction in Study 5, which is absent in Study 4, predominantly results from higher estimates in the ‘neutral-other’ condition in Study 5. Note, however, that a combined 2x2x2 analysis yielded no significant effects of study either as a main effect or as an interaction term suggesting that the difference in results can parsimoniously be considered to be a result of random sampling noise.

## General discussion

Harris and Hahn [28] raised serious doubts over the status of unrealistic optimism, as measured by the traditional comparative method. Their analysis demonstrated that the frequently observed results of unrealistic optimism could be obtained from a population of perfectly rational, unbiased agents. Specifically, they showed how rare events would give rise to negative difference scores, which are taken to suggest that participants—on the whole—see them as less likely to occur to the self than to the average person. For negative events, those most frequently studied, this matches the predictions of an unrealistic optimism hypothesis. In Study 1, we showed that the same negative difference score is also observed for rare positive events, which, of course, should be interpreted as pessimism on the standard unrealistic optimism interpretation, but which is readily predicted by Harris and Hahn’s artifactual account. Seeming pessimism for such classes of events has also been observed previously [40,43,45], suggesting the robustness of this result.

Given the limitations inherent in the standard comparative method, it is difficult to determine whether genuine optimism may simply have been obscured by the statistical artifacts in our data. To test this possibility and provide a sensitive test for optimistic bias, we collected estimates from the same participants of the desirability and frequency of the events. Using this information, we showed that event desirability failed to predict any variance in the comparative optimism data once the influence of statistical artifacts was controlled for via event frequency. Indeed, the pattern in these data trended (weakly) towards pessimism. Studies 2 and 3 attempted to test unrealistic optimism in a more direct manner by providing participants with a fictional scenario that referred to an outcome occurring that would either affect them, or would affect others. There was no evidence that participants estimated the likelihood of a negative event affecting them as less likely than one that only affected others. In Study 3, this result held despite participants generally estimating negative outcomes as more likely than neutral outcomes—the opposite of an optimism bias (replicating the severity effect observed in [20,22–24]. Finally, Studies 4 and 5 utilised the same 2x2 design as Study 3, but moved from fictional scenarios to real outcomes (in which participants—or others—could lose £5 they had been endowed with). Study 4 replicated the results of Study 3. Study 5 failed to replicate the severity effect, but once more there was no evidence for a comparative optimism effect. Studies 2–5 provided the underlying likelihood information to participants in a variety of different ways—some more perceptual than others—thus demonstrating that our results generalize beyond a single paradigm.

The results observed across all five studies, demonstrating no evidence for comparative optimism once the statistical artifacts are controlled for, supports the practical significance of these artifacts following recent skepticism over this issue [34]. As mentioned in the Introduction, however, the current studies do not distinguish between the statistical artifact account and a cognitively focussed egocentrism account. We might have conducted the unconfounded studies (Studies 2–5) in a within-participants design, but suspected that the transparent identical probability required for the self and the other person would have precluded the possibility of observing any effects of optimism. In these clearly chance-based between-participant scenarios, egocentrism would also not appear to predict an optimistic pattern of responding. Consequently, these data were intended to demonstrate clear evidence of a motivational-based unrealistic optimism effect were an effect observed. In the absence of evidence for such an effect, the term ‘optimism’ seems inappropriate to describe the results of studies using the comparative method (c.f. [41]), which may arise as a result of cognitive processes or (and we believe, currently, more parsimoniously) statistical artifacts.

In addition to encouraging skepticism over the practical significance of the artifacts outlined in [28], Shepperd and colleagues [34] highlighted that a critique of this method does not undermine all research on optimism, but is only relevant for research using the comparative methodology. We see Shepperd et al.’s distinction between different potential types of optimism and their methods as an important one that should be maintained in the literature. They are quite correct that the scale artifacts posited in [28] only directly challenge results obtained via the comparative method and thus the phenomenon of unrealistic comparative optimism at the group level (in the terminology of [34]). Our own review of the literature suggests that the evidence for other types of optimism (e.g., absolute optimism or, relatedly, the wishful thinking effect, whereby the desirability of an outcome causes an inflated probability estimate) is likewise overstated (see also, [21,28,41,61,63,69,70]). The current paper is not, however, the appropriate outlet for this debate. The clarification in terminology proposed in [34] is undoubtedly useful and we therefore constrain the implications of the current results as relating to comparative unrealistic optimism. It is important to note, however, that, as recognised in [34], the vast majority of research into optimism addresses this type of comparative optimism, and the critique in [28] thus relates to the majority of research into optimism in general.

As mentioned in the Discussion of Study 1, in light of the flaws identified in the standard comparative method, our view is that a demonstration of comparative unrealistic optimism must employ a method that is not susceptible to the artifacts outlined in [28]. Studies 2–5 introduced such potential methods. Some researchers might argue that the situations are too far removed from consequential, real-world events such as experiencing a heart attack. One thing that differentiates the ‘real-world’ from the ‘experimental world’ of Studies 2–4 is the requirement for information acquisition. Potential events are not typically accompanied by all the information required to estimate their likelihood. Rather, individuals must usually engage in active information acquisition. People might be biased in this process. Indeed, received wisdom suggests that they are (see e.g., [71] for a meta-analytic review). We note, though, that addressing the question of bias in information search will be greatly complicated by the fact that identifying the appropriate normative standard for rational information search has proved somewhat elusive (for debates and questions in this area see, e.g. [72–74]). The focus of the current paper was, however, independent of this question, testing the potential for a direct motivational influence on the estimates people make *from the information they have* (for discussion of the differentiation of these stages of the likelihood estimation process see [23,41]). Participants had *all* the relevant information available to them, but its presentation was sufficiently ambiguous as to enable a biased interpretation—there would have been no scope for the observed severity effect were it not.

Given the complexity associated with investigating events in the real-world, research using unconfounded designs, such as employed in Studies 2–4, is of critical importance in this field. We invite fellow researchers to extend such designs to situations with more extreme outcomes or outcomes upon which substantive decisions must consequently be made. The difficulty, however, with any real-world context is that the estimates participants are required to provide represent the combination of a host of information that is not available to the researcher. A myriad of factors enter into the estimate of “How likely am I to experience a heart attack.” A recognition of how these factors should be combined by the individual is critical to understanding data from such studies. This recognition was the basis for identifying the statistical artifacts proposed in [28]. Thus, in the present paper we employed a more minimal paradigm in which such information was not available to participants. In these situations, we observed no comparative optimism (measured at the group level), despite observing a severity effect in Studies 3 and 4. In the presence of a fundamental critique of previous methods for investigating comparative unrealistic optimism, we see this as the cleanest test to date of the comparative optimism hypothesis.

### Summary

Optimism has been hailed as “the most significant of the cognitive biases” (p. 255 [17]). However, the most prevalent method from which evidence for optimism has been obtained has been shown to be vulnerable to an alternative, artifactual explanation [28]. We conducted five studies testing for unrealistic optimism that take these artifacts into account. Once they were controlled for, we observed no evidence in support of unrealistic optimism whereby participants would perceive negative events as less likely and positive events as more likely to occur to them than others. Our results matched the predictions of the statistical artifact account for unrealistic optimism studies using the comparative method, as well as cognitive accounts such as egocentrism. Evidence for a motivation-based, and therefore truly optimistic account of the data using the comparative method, was thus not found. These data are parsimoniously explained as either stemming from non-optimistic egocentric cognitive processes (e.g., [45]) or the statistical artifacts inherent in the methods used.

## Supporting information

S1 Table Data reproduced from columns 1, 2 and 4 of Klar and Ayal (Table 1) [55]. (DOCX)

S2 Table Comparative responses for common negative events. All events were rated as significantly negative by participants. Asterisks denote responses significantly different from zero (comparative judgments), and 50% (frequency judgments). Four of the five events rated as significantly common received significantly positive comparative ratings, in line with the predictions of the statistical account.
(DOCX)

## References

[1] Weinstein ND. Unrealistic optimism about future life events. J Pers Soc Psychol. 1980;39(5):806–20.

[2] Burger JM, Burns L. The illusion of unique invulnerability and the use of effective contraception. Personal Soc Psychol Bull. 1988;14:264–70. 10.1177/0146167288142005 30045472

[3] Campbell J, Greenauer N, Macaluso K, End C. Unrealistic optimism in internet events. Comput Human Behav. 2007;23:1273–84.

[4] Harris DM, Guten S. Health-Protective Behavior: An exploratory study. J Health Soc Behav. 1979;20:17–29. 438490

[5] Harris P, Middleton W. The illusion of control and optimism about health: On being less at risk but no more in control than others. Br J Soc Psychol. 1994;33:369–86. 7842244 10.1111/j.2044-8309.1994.tb01035.x

[6] Hevey D, French DP. Comparative optimism for severity of negative health outcomes. Psychol Health Med [Internet]. 2012;17(4):417–26. Available from: http://www.ncbi.nlm.nih.gov/pubmed/22111753 10.1080/13548506.2011.613940 22111753

[7] Joshi MS, Carter W. Unrealistic Optimism: East and West? Front Psychol [Internet]. 2013;4(February):1–15. Available from: http://journal.frontiersin.org/article/10.3389/fpsyg.2013.00006/abstract 10.3389/fpsyg.2013.00006 PMC3570981 23407689

[8] Lek Y, Bishop GD. Perceived vulnerability to illness threats: The role of disease type, risk factor perception and attributions. Psychol Heal. 1995;10:205–17.

[9] Otten W, van der Pligt J. Context effects in the measurement of comparative optimism in probability judgments. J Soc Clin Psychol. 1996;15:80–101.

[10] Perloff LS, Fetzer BK. Self-other judgments and perceived vulnerability to victimization. J Pers Soc Psychol. 1986;50:502–10.

[11] Regan PC, Snyder M, Kassin SM. Unrealistic optimism, self-enhancement or person positivity. Personal Soc Psychol Bull. 1995;21:1073–82.

[12] Weinstein ND, Lachendro E. Egocentrism as a Source of Unrealistic Optimism. Personal Soc Psychol Bull [Internet]. 1982 6 1 [cited 2013 Oct 18];8(2):195–200. Available from: http://psp.sagepub.com/cgi/doi/10.1177/0146167282082002

[13] Weinstein ND. Why it won’t happen to me: Perceptions of risk factors and susceptibility. Heal Psychol. 1984;3:431–57. 10.1037//0278-6133.3.5.431 6536498

[14] Weinstein ND. Unrealistic optimism about susceptibility to health problems: Conclusions from a community-wide sample. J Behav Med. 1987;10:481–500. 3430590 10.1007/BF00846146

[15] Weinstein ND, Klein WM. Resistance of personal risk perceptions to debiasing interventions. Heal Psychol. 1995;14:132–40. 10.1037//0278-6133.14.2.132 7789348

[16] Whitley BE, Hern AL. Perceptions of vulnerability to pregnancy and the use of effective contraception. Personal Soc Psychol Bull. 1991;17:104–10.

[17] Kahneman D. Thinking, fast and slow. London, UK: Penguin; 2011.

[18] Sharot T. The optimism bias: Why we’re wired to look on the bright side. London, UK: Constable & Robinson Ltd.; 2012.

[19] Sample I. How the brain filters bad news [Internet]. Guardian. 2012. http://www.guardian.co.uk/science/blog/2012/sep/25/how-brain-filters-bad-news?newsfeed=true

[20] Vosgerau J. How prevalent is wishful thinking? Misattribution of arousal causes optimism and pessimism in subjective probabilities. J Exp Psychol Gen. 2010;139:32–48. 10.1037/a0018144 20121311

[21] de Molière L, Harris AJL. Conceptual and direct replications fail to support the stake-likelihood hypothesis as an explanation for the interdependence of utility and likelihood judgments. J Exp Psychol Gen. 2016;145:e13–26. 10.1037/xge0000124 26974210

[22] Bilgin B. Organizational Behavior and Human Decision Processes Losses loom more likely than gains: Propensity to imagine losses increases their subjective probability. Organ Behav Hum Decis [Internet]. Elsevier Inc.; 2012; 10.1016/j.obhdp.2012.03.008

[23] Harris AJL, Corner A, Hahn U. Estimating the probability of negative events. Cognition. 2009;110:51–64. 10.1016/j.cognition.2008.10.006 19036359

[24] Risen JL, Gilovich T. Another look at why people are reluctant to exchange lottery tickets. J Pers Soc Psychol. 2007;93:12–22. 10.1037/0022-3514.93.1.12 17605585

[25] Harris AJL, Corner A. Communicating environmental risks: Clarifying the severity effect in interpretations of verbal probability expressions. J Exp Psychol Learn Mem Cogn. 2011;37:1571–8. 10.1037/a0024195 21767064

[26] Taylor SE, Brown JD. Illusion and well-being: A social psychological perspective on mental health. Psychol Bull. 1988;103:193–210. 3283814

[27] Weinstein ND. Unrealistic optimism about susceptibility to health problems. J Behav Med. 1982;5:441–60. 7154065 10.1007/BF00845372

[28] Harris AJL, Hahn U. Unrealistic optimism about future life events: A cautionary note. Psychol Rev. 2011;118:135–54. 10.1037/a0020997 21058872

[29] Covey JA, Davies ADM. Are people unrealistically optimistic? It depends how you ask them. Br J Health Psychol. 2004;9:39–49. 10.1348/135910704322778713 15006200

[30] Klar Y, Medding A, Sarel D. Nonunique invulnerability: Singular versus distributional probabilities and unrealistic optimism in comparative risk judgments. Organ Behav Hum Decis Process. 1996;67:229–45.

[31] Price PC, Pentecost HC, Voth RD. Perceived event frequency and the optimistic bias: Evidence for a two-process model of personal risk judgments. J Exp Soc Psychol. 2002;38:242–52.

[32] Welkenhuysen M, Evers-kiebooms G, Van Den Berghe H. Unrealistic optimism and genetic risk. Psychol Heal. 1996;(11):479–92.

[33] Statistics O for N. Registrations of cancer diagnosed in 1994–1997, England and Wales. Heal Stat Q. 2000;7:71–82.

[34] Shepperd JA, Klein WMP, Waters EA, Weinstein ND. Taking Stock of Unrealistic Optimism. Perspect Psychol Sci. 2013;8:395–411. 10.1177/1745691613485247 26045714 PMC4451212

[35] Fox CR, Hadar L. “Decisions from experience” = sampling error + prospect theory: Reconsidering Hertwig, Barron, Weber & Erev (2004). Judgm Decis Mak. 2006;1:159–61.

[36] Hertwig R, Barron G, Weber EU, Erev I. Decisions from experience and the effect of rare events in risky choice. Psychol Sci. 2004;15:534–7. 10.1111/j.0956-7976.2004.00715.x 15270998

[37] Ungemach C, Chater N, Stewart N. Are probabilities overweighted or underweighted when rare outcomes are experienced (rarely)? Psychol Sci. 2009;20:473–9. 10.1111/j.1467-9280.2009.02319.x 19399978

[38] Hardman D. Judgment and decision making: Psychological perspectives. Chichester, UK: BPS Blackwell; 2009.

[39] Kahneman D, Tversky A. On the psychology of prediction. Psychol Rev. 1973;80:237–51.

[40] Moore DA, Small D. When it is rational for the majority to believe that they are better than average In: Krueger JI, editor. Rationality and Social Responsibility: Essays in honor of Robyn Dawes. New York, NY: Psychology Press; 2008 p. 141–74.

[41] Hahn U, Harris AJL. What does it mean to be biased: Motivated reasoning and rationality. Psychol Learn Motiv. 2014;61:41–102.

[42] Chambers JR, Windschitl PD, Suls J. Egocentrism, event frequency, and comparative optimism: when what happens frequently is “more likely to happen to me”. Pers Soc Psychol Bull [Internet]. 2003 11 [cited 2013 Oct 18];29(11):1343–56. Available from: http://www.ncbi.nlm.nih.gov/pubmed/15189574 10.1177/0146167203256870 15189574

[43] Kruger J, Burrus J. Egocentrism and focalism in unrealistic optimism (and pessimism). J Exp Soc Psychol. 2004;40:332–40.

[44] Chang EC, Asakawa K, Sanna LJ. Cultural variations in optimistic and pessimistic bias: Do easterners really expect the worst and westerners really expect the best when predicting future life events? J Pers Soc Psychol. 2001;81:476–91. 11554648

[45] Chambers JR, Windschitl PD, Suls J. Egocentrism, event frequency, and comparative optimism: When what happens frequently is “more likely to happen to me.” Personal Soc Psychol Bull. 2003;29:1343–56. 10.1177/0146167203256870 15189574

[46] Pyszczynski T, Holt K, Greenberg J. Depression, self-focused attention, and expectancies for positive and negative future life events for self and others. J Pers Soc Psychol. 1987;52:994–1001. 3585706 10.1037//0022-3514.52.5.994

[47] Hoorens V, Smits T, Shepperd J a. Comparative optimism in the spontaneous generation of future life-events. Br J Soc Psychol [Internet]. 2008;47(3):441–51. Available from: http://doi.wiley.com/10.1348/014466607X236023 10.1348/014466607X236023 17723156

[48] Walter FM, Emery J. Perceptions of family history across common diseases: A qualitative study in primary care. Fam Pract. 2006;23:472–80. 10.1093/fampra/cml006 16608871

[49] Van der Velde FW, Hooykas C, Van der Pligt J. Risk perception and behavior: Pessimism, realism, and optimism about AIDS-related health behavior. Psychol Heal. 1992;6:23–38.

[50] Zakay D. The influence of perceived event’s controllability on its subjective occurrence probability. Psychol Rec. 1984;34:233–40.

[51] Zakay D. The relativity of unrealistic optimism. Acta Psychol (Amst). 1996;93:121–31. 8826792 10.1016/0001-6918(96)00025-x

[52] Klein CTF, Helweg-Larsen M. Perceived control and the optimistic bias: A meta-analytic review. Psychol Health. 2002;17:437–46.

[53] Windschitl PD, Rose JP, Stalkfleet MT, Smith AR. Are people excessive or judicious in their egocentrism? A modeling approach to understanding bias and accuracy in people’s optimism. J Pers Soc Psychol. 2008;95(2):253–73. 10.1037/0022-3514.95.2.253 18665701

[54] Rose JP, Endo Y, Windschitl PD, Suls J. Cultural Differences in Unrealistic Optimism and Pessimism: The Role of Egocentrism and Direct Versus Indirect Comparison Measures. Personal Soc Psychol Bull [Internet]. 2008;34(9):1236–48. Available from: http://psp.sagepub.com/cgi/doi/10.1177/0146167208319764 10.1177/0146167208319764 18587057

[55] Klar Y, Ayal S. Event frequency and comparative optimism: Another look at the indirect elicitation method of self-others risks. J Exp Soc Psychol. 2004;40:805–14.

[56] Chambers JR, Windschitl PD. Biases in social comparative judgments: The role of nonmotivated factos in above-average and comparative-optimism effects. Psychol Bull. 2004;130:813–38. 10.1037/0033-2909.130.5.813 15367082

[57] Howell D C. Statistical methods for psychology. 4th ed Belmont, CA: Duxbury Press; 1997.

[58] Lorch RFJ, Myers JL. Regression analyses of repeated measures data in cognitive research. J Exp Psychol Learn Mem Cogn. 1990;16:149–57. 2136750 10.1037//0278-7393.16.1.149

[59] Dunning D, Story AL. Depression, realism, and the overconfidence effect: Are the sadder wiser when predicting future actions and events? J Pers Soc Psychol. 1991;61:521–32. 1960645 10.1037//0022-3514.61.4.521

[60] Tversky a, Kahneman D. Judgment under Uncertainty: Heuristics and Biases. Science [Internet]. 1974 9 27;185(4157):1124–31. Available from: http://www.ncbi.nlm.nih.gov/pubmed/17835457 10.1126/science.185.4157.1124 17835457

[61] Bar-Hillel M, Budescu D. The elusive wishful thinking effect. Think Reason. 1995;1:71–103.

[62] Kunda Z. The case for motivated reasoning. Psychol Bull. 1990;108:480–98. 2270237 10.1037/0033-2909.108.3.480

[63] Harris AJL. Understanding the coherence of the severity effect and optimism phenomena: Lessons from attention. Conscious Cogn. 10.1016/j.concog.2016.10.014 27866703

[64] Rouder JN, Speckman PL, Sun D, Morey RD, Iverson G. Bayesian t tests for accepting and rejecting the null hypothesis. Psychon Bull Rev. 2009;16:225–37. 10.3758/PBR.16.2.225 19293088

[65] Morey RD, Rouder JN. Bayes factor approaches for testing interval null hypotheses. Psychol Methods [Internet]. 2011 12 [cited 2013 Jun 18];16(4):406–19. Available from: http://www.ncbi.nlm.nih.gov/pubmed/21787084 10.1037/a0024377 21787084

[66] Miller LC, Murphy R, Buss AH. Consciousness of body: Private and public. J Pers Soc Psychol. 1981;41:397–406.

[67] Rouder JN, Morey RD, Speckman PL, Province JM. Default Bayes factors for ANOVA designs. J Math Psychol [Internet]. Elsevier Inc.; 2012 10 [cited 2013 Jun 12];56(5):356–74. Available from: http://linkinghub.elsevier.com/retrieve/pii/S0022249612000806

[68] Peck J, Shu SB. The effect of mere touch on perceived ownership. J Consum Res. 2009;36:434–47.

[69] Bar-Hillel M, Budescu D V., Amar M. Predicting World Cup results: Do goals seem more likely when they pay off? Psychon Bull Rev [Internet]. 2008 4 1 [cited 2012 Apr 11];15(2):278–83. Available from: http://pbr.psychonomic-journals.org/cgi/doi/10.3758/PBR.15.2.278 10.3758/pbr.15.2.278 18488640

[70] Krizan Z, Windschitl PD. The influence of outcome desirability on optimism. Psychol Bull. 2007;133:95–121. 10.1037/0033-2909.133.1.95 17201572

[71] Hart W, Albarracín D, Eagly AH, Brechan I, Lindberg MJ, Merrill L. Feeling validated versus being correct: a meta-analysis of selective exposure to information. Psychol Bull [Internet]. 2009 7 [cited 2013 Nov 11];135(4):555–88. Available from: http://www.ncbi.nlm.nih.gov/pubmed/19586162 10.1037/a0015701 PMC4797953 19586162

[72] Oaksford M, Chater N. A rational analysis of the selection task as optimal data selection. Psychol Rev. 1994;101:608–31.

[73] Nelson JD. Finding Useful Questions: On Bayesian Diagnosticity, Probability, Impact, and Information Gain. Psychol Rev. 2005;112(4):979–999. 10.1037/0033-295X.112.4.979 16262476

[74] Klayman J, Ha Y. Confirmation, disconfirmation, and information in hypothesis testing. 1987;94(2):211–28.

