# Correction: Unrealistic comparative optimism: An unsuccessful search for evidence of a genuinely motivational bias

**DOI:** 10.1371/journal.pone.0175245

**Published:** 2017-04-03

**Authors:** The PLOS ONE Staff

A portion of the figure legend for Table 1 is incorrectly displayed in the text of the Results section in the second and third paragraph of the section headed “Comparing the effects of perceived frequency and event valence.” The text in question is “Four of Weinstein’s original items…according to participants’ subjective ratings.” Please see the complete, correct Table 1 and caption here. The publisher apologizes for the error.

**Table 1:** ‘Unrealistic optimism’ for future life events.

	Mean comparative judgment of own chances vs others' chances	No. of optimistic responses divided by no. of pessimistic responses	Mean perceived frequency
Event			
Positive events			
Own own home	1.28 ***	11.17 ***	72.35
Like job after university	0.65 ***	2.39 ***	52.78
Starting salary >£20,000	0.4 **	2.93 ***	53.16
Not spend a night in hospital in 5 years	0.25 ns.	1.39 ns.	53.38
Have a mentally gifted child	0.16 ns.	1.67 ns.	19.62
Visit Amazonian rainforest	-0.12 ns.	0.98 ns.	10.31
Home's value doubles in 5 years	-0.19 ns.	0.80 ns.	25.50
Live past 90 years old	*-0*.*45* **	0.73 ns.	22.58
Maintain a constant weight for 10 years	*-0*.*67* **	0.73 ns.	32.86
Graduate with a first	*-0*.*69* **	*0*.60 *	25.71
Work recognised with an award	*-0*.*74* ***	*0*.*43* ***	11.39
Last whole winter without being ill	*-0*.*74* ***	*0*.*48* ***	28.91
Receive good job offer before graduating	*-0*.*83* ***	*0*.*24* ***	27.05
Starting salary >£30,000	*-0*.*84* ***	*0*.*31* ***	26.20
Achievements acknolwedged in national press	*-0*.*97* ***	*0*.*27* ***	7.53
Earn >£80,000 in 10 years time	*-1*.*08* ***	*0*.*22* ***	16.24
Nationwide recognition within profession	*-1*.*26* ***	*0*.*23* ***	7.11
Starting salary >£40,000	*-1*.*38* ***	*0*.*16* ***	13.28
Marry a millionaire	*-1*.*52* ***	*0*.*19* ***	4.01
Negative events			
Marry a film star	-1.84 ***	9.43 ***	1.10
Contract AIDS	-1.75 ***	8.86 ***	3.33
Divorced within 5 years of marriage	-1.25 ***	3.93 ***	32.56
Lung cancer	-1.21 ***	4.18 ***	12.57
Have a drinking problem	-0.88 ***	2.15 ***	13.35
Be sued	-0.82 ***	3.06 ***	10.51
Be fired from a job	-0.66 ***	2.83 ***	22.28
Heart attack before 40	-0.65 ***	2.27 ***	6.09
Be unable to have children	-0.07 ns.	0.96 ns.	11.20
Heart attack	0.03 ns.	1.03 ns.	17.48
Have car stolen	0.03 ns.	0.79 ns.	20.19
Out of work for 6 months	0.04 ns.	1.00 ns.	37.27
Be the victim of a mugging	0.09 ns.	0.71 ns.	24.25
Buy a car that turns out to be terrible	0.19 ns.	0.63 ns.	39.35
Realise chose the wrong career	0.20 ns.	0.58 *	34.66
Be the victim of burglary	0.22 ns.	*0*.*39* ***	40.74
Be in bed ill for 2 or more days in a year	0.29 ns.	0.77 ns.	67.06
Forced to take an unattractive job	0.32 *	*0*.*43* ***	49.78
Cancer	0.34 *	0.62 ns.	32.31
Break a bone	0.38 *	0.70 ns.	48.39
Injured in a road accident	*0*.*41* ***	*0*.*26* ***	26.92

ns. = nonsignificant. Italicised means represent significant pessimism.

*p < .05.

**p < .01.

***p < .001. Four of Weinstein's original items were not included in this study. These were: "Dropping out of college" (to reduce any extra variance introduced as a result of participants being both first and second year students). "Decayed tooth extracted" and "Having gum problems" (as such events may not be future events for some of the sample), and "attempting suicide" (for ethical reasons). Events are classified here as positive or negative according to participants' subjective ratings.
