# Supplementary material for: Unrealistic comparative optimism: An unsuccessful search for evidence of a genuinely motivational bias
Source: PLoS One. 2017 Mar 9;12(3):e0173136. doi: 10.1371/journal.pone.0173136 (PMC5344342; doi:10.1371/journal.pone.0173136)
Supplement: S1 Table — (DOCX) [file pone.0173136.s001.docx]

| Event | Average risk estimates | Personal risk estimates | Ratio (others/self) |
| --- | --- | --- | --- |
| Limb amputated | 16.93 | 11.80 | 1.43 |
| Suicide | 15.06 | 3.68 | 4.09 |
| House fire | 20.03 | 16.45 | 1.22 |
| AIDS | 19.60 | 8.92 | 2.20 |
| Gum disease | 44.16 | 41.08 | 1.07 |
| Hearing loss | 14.18 | 9.38 | 1.51 |
| Death by car crash | 30.37 | 26.07 | 1.16 |
| Sued | 31.12 | 19.95 | 1.56 |
| Cancer | 26.11 | 19.12 | 1.37 |
| Alcoholism | 22.99 | 4.57 | 5.03 |
| Heart attack | 29.88 | 16.18 | 1.85 |
| Car stolen | 33.45 | 25.86 | 1.29 |
| Death before 65 years of age | 33.25 | 28.20 | 1.18 |
| Drops out of school college | 29.17 | 15.54 | 1.88 |
| Depression | 22.86 | 14.61 | 1.56 |
| Burglarized | 29.80 | 23.78 | 1.25 |
| Fired from job | 46.59 | 36.06 | 1.29 |
| Bad career choice | 51.93 | 37.04 | 1.40 |
| Broken bone | 31.29 | 25.77 | 1.21 |
| Divorce | 43.24 | 12.76 | 3.39 |

**S1 Table. Data reproduced from columns 1, 2 and 4 of Klar and Ayal (Table 1)[55].**
