# Supplementary material for: Unrealistic comparative optimism: An unsuccessful search for evidence of a genuinely motivational bias
Source: PLoS One. 2017 Mar 9;12(3):e0173136. doi: 10.1371/journal.pone.0173136 (PMC5344342; doi:10.1371/journal.pone.0173136)
Supplement: S2 Table — All events were rated as significantly negative by participants. Asterisks denote responses significantly different from zero (comparative judgments), and 50% (frequency judgments). Four of the five events rated as significantly common received significantly positive comparative ratings, in line with the predictions of the statistical account. (DOCX) [file pone.0173136.s002.docx]

**S2 Table. Comparative responses for common negative events.**

|  |  |  |  | Mean comparative judgment of own chances vs others' chances | |  |
| --- | --- | --- | --- | --- | --- | --- |
|  |  |  |  |  |  | Mean perceived frequency |
|  |  |  |  |  |  |  |
| Event | | | |  |  |  |
|  |  |  |  |  |  |  |
|  |  |  |  |  |  |  |
| Feel stress at some stage of your | | |  | 1.14*** | | 89.72*** |
| university studies | |  |  |  |  |  |
|  |  |  |  |  | |  |
| Gain half a stone in weight by the time | | | | 0.71*** | | 70.94*** |
| you are 50 |  |  |  |  |  |  |
|  |  |  |  |  |  |  |
| Attend the funeral of a loved one in | | | | 0.61*** | | 67.70*** |
| the next 20 years | |  |  |  | |  |
|  |  |  |  |  |  |  |
| Go to have a shower only to discover | | | | 0.57*** | | 73.35*** |
| that the hot water has run out | | |  |  |  |  |
|  |  |  |  |  |  |  |
| Have your heart broken at some stage | | | | 0.31 ns. | | 69.26*** |
| in your life |  |  |  |  |  |  |
|  |  |  |  |  |  |  |
| Have painful treatment at the dentist | | | | -0.01 ns. | | 53.60 ns. |
| at some stage in your life | | |  |  |  |  |
|  |  |  |  |  |  |  |
| Receive a speeding ticket at some | | |  | -0.17 ns. | | 46.75 ns. |
| stage in your life | |  |  |  |  |  |
|  |  |  |  |  |  |  |
| ns. = nonsignificant. | | | | | | |
| **p* < .05. ***p* < .01 ****p* <.001. | | |  |  |  |  |
